# Investigation of circulating metabolites associated with breast cancer risk by untargeted metabolomics: a case–control study nested within the French E3N cohort

**DOI:** 10.1038/s41416-021-01304-1

**Published:** 2021-03-15

**Authors:** Elodie Jobard, Laure Dossus, Laura Baglietto, Marco Fornili, Lucie Lécuyer, Francesca Romana Mancini, Marc J. Gunter, Olivier Trédan, Marie-Christine Boutron-Ruault, Bénédicte Elena-Herrmann, Gianluca Severi, Joseph A. Rothwell

**Affiliations:** 1 Univ Lyon, CNRS, Université Claude Bernard Lyon 1, ENS de Lyon, Institut des Sciences Analytiques, UMR 5280, Villeurbanne, France; 2 Université de Lyon, Centre Léon Bérard, Département d’Oncologie Médicale, Lyon, France; 3 Nutrition and Metabolism Section, International Agency for Research on Cancer, Lyon, France; 4 Department of Clinical and Experimental Medicine, University of Pisa, Pisa, Italy; 5 Université Paris-Saclay, UVSQ, Univ. Paris-Sud, Inserm, Gustave Roussy, Exposome and Heredity Team, Centre for Epidemiology and Population Health, Villejuif, France; 6 Univ Grenoble Alpes, CNRS, INSERM, IAB, Allée des Alpes, Grenoble, France; 7 Department of Statistics, Computer Science and Applications “G. Parenti” (DISIA), University of Florence, Firenze, Italy

**Keywords:** Breast cancer, Breast cancer, Predictive markers, Epidemiology, Lifestyle modification

## Abstract

**Background:**

Perturbations in circulating metabolites prior to a breast cancer diagnosis are not well characterised. We aimed to gain more detailed knowledge to help understand and prevent the disease.

**Methods:**

Baseline plasma samples from 791 breast cancer cases and 791 matched controls from the E3N (EPIC-France) cohort were profiled by nuclear magnetic resonance (NMR)-based untargeted metabolomics. Partial least-squares discriminant analysis (PLS-DA) models were built from NMR profiles to predict disease outcome, and odds ratios and false discovery rate (FDR)-adjusted CIs were calculated for 43 identified metabolites by conditional logistic regression.

**Results:**

Breast cancer onset was predicted in the premenopausal subgroup with modest accuracy (AUC 0.61, 95% CI: 0.49–0.73), and 10 metabolites associated with risk, particularly histidine (OR = 1.70 per SD increase, FDR-adjusted CI 1.19–2.41), N-acetyl glycoproteins (OR = 1.53, FDR-adjusted CI 1.18–1.97), glycerol (OR = 1.55, FDR-adjusted CI 1.11–2.18) and ethanol (OR = 1.44, FDR-adjusted CI 1.05–1.97). No predictive capacity or significant metabolites were found overall or for postmenopausal women.

**Conclusions:**

Perturbed metabolism compared to controls was observed in premenopausal but not postmenopausal cases. Histidine and NAC have known involvement in inflammatory pathways, and the robust association of ethanol with risk suggests the involvement of alcohol intake.

## Background

Breast cancer is the most common cancer in women and accounts for around 25% of all female cancer cases worldwide.^1^ Alcohol intake, adult obesity and greater birthweight and height are reported to increase risk, while physical activity, breastfeeding and calcium intake have been linked with a decreased risk in population studies.^2–4^ Breast cancer is also a heterogeneous disease and risk factors vary between oestrogen receptor-positive and negative tumours^5^ and according to menopausal status.^6^

Knowledge of pre-diagnostic metabolism can potentially help identify population subgroups at greater risk and provide insight into the mechanisms of early carcinogenesis. A small number of studies have employed metabolomics previously to estimate associations prospectively between breast cancer risk and plasma or serum metabolite concentrations.^7–13^ Diverse study designs and analytical platforms have been employed, and although some broad common conclusions have been reached, such as the inverse associations between fatty acid-derived metabolites and breast cancer risk, important issues remain to be addressed. Firstly, few of these studies have presented results by menopausal status, which may be an important determinant of both normal and pathological metabolic conditions. Secondly, alcohol intake is a likely risk factor for breast cancer, but most previous studies have relied on participant self-reported assessment. Although previous studies controlled for self-reported alcohol intake, few were able to include a biomarker surrogate for additional validation.

In this study, we test plasma metabolite associations with breast cancer risk using one of the largest metabolomics studies to date on the disease, in the French E3N cohort. Our study benefited from untargeted NMR metabolomics data that included the measurement of proxies of systemic inflammation (N-acetyl glycoproteins) and recent alcohol intake (free plasma ethanol), as well as a range of other endogenous metabolites. We aimed firstly to determine if full untargeted NMR plasma profiles were able to distinguish pre-diagnostic breast cancer cases from controls using multivariate predictive models, and secondly to test metabolite associations prospectively with breast cancer risk. Knowledge of how plasma metabolism is perturbed prior to a diagnosis may help disentangle the complex web of risk factors and translate into more effective disease prevention strategies.

## Methods

### Study design and sample collection

The present study is based on a case–control study nested within the *Etude Epidémiologique auprès de femmes de la MGEN (Mutuelle Générale de l’Education Nationale)* cohort (E3N), a French multicentre prospective study designed to investigate risk factors for cancer and major non-communicable diseases in women. The cohort comprised nearly 100,000 women aged 40–65 years insured through a national health system and enroled from 1990 after returning baseline self-administered questionnaires.^14,15^ Every 2–3 years after baseline, follow-up questionnaires were collected to update information about diet, lifestyle characteristics and medical events. These included detailed food-frequency questionnaires that allowed the calculation of alcohol intake in g/day. Blood samples were collected from around a quarter of all participants between 1994 and 1999. The E3N cohort was granted ethical approval by the French National Commission for Computed Data and Individual Freedom (*Commission Nationale de l’Informatique et des Libertés*) and all E3N participants provided written consent for the use of their blood samples and all data. E3N is the French component of the European Prospective Investigation into Cancer and Nutrition (EPIC), a collaborative study of over 500,000 participants in 10 countries coordinated by the International Agency for Research on Cancer, Lyon, France.

### Case ascertainment and matching

Participants of E3N were asked to declare any new cancer event in periodic follow-up questionnaires. These reports were then investigated and validated by collecting pathological reports or clinical records from doctors. Tumour characteristics, such as stage, behaviour, histological subtype and hormone receptor status, were extracted from the reports. Incident breast cancer cases (*n* = 812) with available blood samples at baseline were identified. Each case was matched to a control who was free of cancer at the time of diagnosis. Matching factors were age at blood collection (±1 year), *département* (county) of residence (or collection centre), blood collection date (±9 months), menopausal status at blood collection date (pre- or post-menopausal) and fasting status (yes or no). After the exclusion of 7 breast cancer cases with no eligible control, 1610 plasma samples (805 cases and 805 matched controls) were retained.

### Sample preparation and analysis

Blood samples were collected, processed and stored as previously described.^16^ To obtain plasma fractions, blood samples were recovered from citrate collection tubes and centrifuged at 1500 × *g* for 20 min. The samples were then stored in liquid nitrogen at −196 °C until laboratory analysis. To check data quality and reproducibility, two quality control plasma samples (QC) collected from one healthy donor were prepared in parallel with experimental samples. These were placed at the beginning and end of each sample rack to evaluate analytical variability.

One-dimensional ^1^H-NMR spectra were acquired on a Bruker Avance III spectrometer (Bruker, Rheinstetten, Germany) operating at 600.55 MHz and equipped with a temperature-controlled automatic sample changer and 5-mm TCI cryo probe. Standard ^1^H-NMR pulse sequences, NOESY and CPMG with water pre-saturation, were applied to samples to generate raw spectra (Supplementary Fig. S1). The NOESY mixing time was set to 100 ms and the total CPMG filter to 80 ms for efficient attenuation of the lipid NMR signals. These spectra were manually phased and baseline-corrected before being imported into Bruker Amix software for processing. Spectra were reduced, over a chemical shift range of 0.5–9.0 ppm, to 8500 bins, each of which was integrated as a separate variable.

Two-dimensional NMR spectra were additionally acquired from one case and one control sample to assign NMR signals observed in the ^1^H one-dimensional profiles to metabolite identities. Fifty-six identities were assigned from interactive analysis of this dataset and reference to NMR shift knowledge bases (Supplementary Table S1). For metabolite quantification, NMR chemical shift regions were grouped into 243 buckets that corresponded to reconstructions of peak entities. Clusters of variables corresponding to the same metabolite were then summed to give a single intensity, resulting in 43 measurements that corresponded to distinct metabolites or metabolite classes. Full details are given in Supplementary Information.

### Statistical analysis

After the exclusion of 28 participants for which spectra did not meet quality control checks, 1582 remained for statistical analysis. Relative standard deviations (RSD) were calculated for each chemical shift region as a check of analytical reproducibility, and the PC-PR2 method was employed to assess the effect of different variables upon metabolomics data.^17^ Predictive models for sample classification were fit using partial least-squares discriminant analysis (PLS-DA) based on full untargeted NMR profiles. Models were fit for all participants, by menopausal status at blood collection (*n* = 354 and 1218 for pre- and post-menopausal, respectively), age category at diagnosis (over or under 55 years), fasting status at blood collection (yes or no) and time to diagnosis (within 2 or 5 years of blood collection).

NMR variables were transformed to the residuals of a linear model of metabolite intensity on blood collection centre, week of laboratory analysis, biobank storage time, waiting time to sample fractionation and fasting status.^18^ This matrix of residuals was used to fit a partial least-squares discriminant analysis (PLS-DA) model with case–control status as a binary response. Models were trained and refined on a random 75% of these data and tested on the remainder, and this final model used to predict case–control status for the test observations. Accuracy, as receiver-operating characteristic (ROC) area under the curve (AUC), was used to assess model performance. Analyses were performed using R statistical software, version 3.5.2 and PLS-DA models were fit using the caret package.^19^

Odds ratios (OR) and 95% confidence intervals were then calculated for each of the 43 annotated metabolites using conditional logistic regression. The highest and lowest 1% of intensities were first excluded for each metabolite and data were modelled as continuous variables with odds ratios corresponding to a one-SD increase in relative concentration. Models were adjusted for sample waiting time before fractionation, BMI, diabetes status, sample-storage time, waist-to-hip ratio, daily alcohol intake (g/day) and duration of use of menopause hormonal treatment at blood collection. To account for multiple testing, *P*-values were adjusted using the false discovery rate (FDR) procedure and the significance threshold set at 0.05. For those associations that were significant by these criteria, FDR-adjusted confidence intervals (CI) for ORs were also calculated using the method of Benjamini and Yekutieli.^20^

The relationship between reported alcohol intake and plasma ethanol was investigated on a continuous basis by Pearson correlation and for quartiles of alcohol intake by the Kruskal–Wallis one-way analysis of variance. As a sensitivity analysis and further investigation of alcohol intake in relation to breast cancer, the premenopausal metabolite risk models were repeated treating plasma ethanol concentration as a measure of exposure to alcohol, adjusting for this variable in all other metabolite models.

A sensitivity analysis was carried out excluding non-fasting matched pairs overall and by menopausal status. For the premenopausal subgroup only, further sensitivity analyses were performed adjusting for lifetime alcohol intake pattern and excluding cases diagnosed in the first two years after blood collection and their corresponding controls.

## Results

### Participant and tumour characteristics

**Fig. 1:**
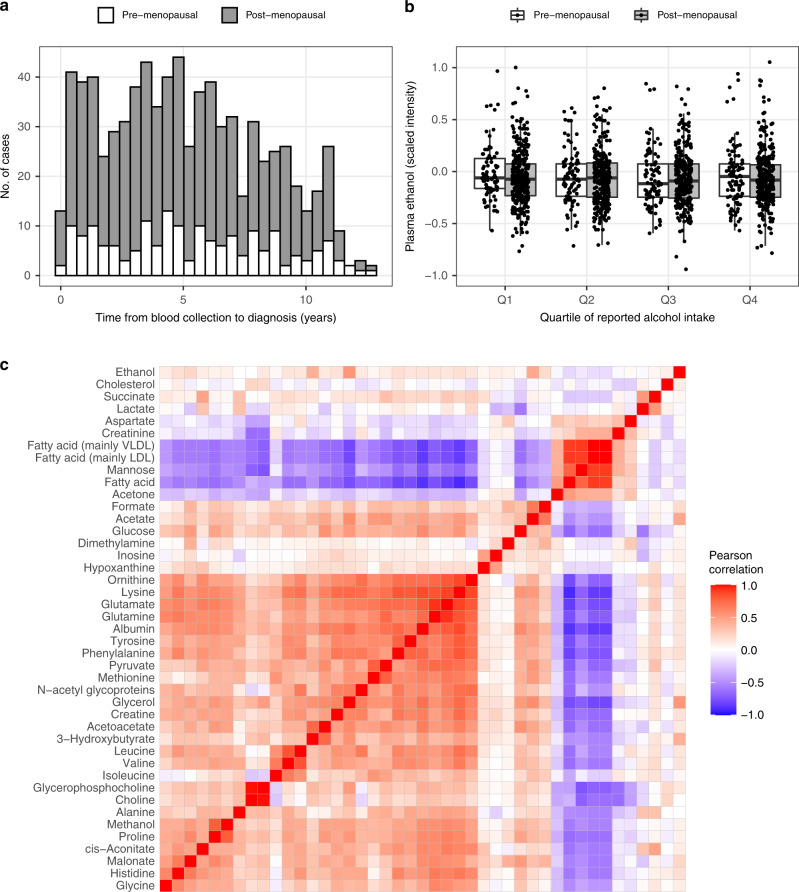
Descriptive analysis of participant and metabolite data. **a** Distribution of follow-up times to diagnosis. **b** Relationship between reported alcohol intake and plasma ethanol concentrations. **c** Plasma metabolites identified and their correlations. No significant association was found between quartile of alcohol intake and plasma ethanol concentration (Kruskal–Wallis *P* > 0.05 for pre- and postmenopausal groups). Correlations were calculated from fasted participants only and metabolites are ordered by hierarchical cluster as determined by Ward’s method.

Baseline characteristics of the metabolomics study participants are shown in Table 1 and Supplementary Table S2A. Most cases (77%) were post-menopausal at blood collection and the median time to diagnosis was 4.75 years (range 0.01–12.67 years) (Fig. 1a). Cases were more frequently users of menopause hormone therapy at the time of blood collection. Most tumours were invasive ductal carcinomas and were oestrogen receptor (ER) positive (Supplementary Table S2B). Cases who were pre- and post-menopausal at blood collection did not differ in tumour subtype, behaviour, grade or ER status, and differed slightly only in the proportion of progesterone receptor- positive cases. Only 8 cases that were pre-menopausal at blood collection were reported to be post-menopausal at breast cancer diagnosis.

**Table 1:** Characteristics of the breast cancer metabolomics case–control study participants.

Baseline characteristic	Mean ± SD or frequency (%)		
	Controls (*N* = 791)	Cases (*N* = 791)	*P*-value^a^
Age at blood collection (years)	56.8 ± 6.6	56.8 ± 6.6	–
Menopausal status at blood collection			
Pre-menopausal	180 (22.5)	179 (22.5)	–
Post-menopausal	611 (77.5)	612 (77.5)	
Fasting status at blood collection			
Fasting	287 (36.3)	287 (36.3)	–
Non-fasting	504 (63.7)	504 (63.7)	
Follow-up time to cancer diagnosis			
5 years or less	–	412 (52.1)	–
More than 5 years	–	379 (47.9)	
BMI (kg/m^2^)			
Underweight or normal (<25)	538 (68.3)	564 (71.4)	0.27
Overweight^25–30^	204 (25.9)	177 (22.4)	
Obese (≥30)	46 (5.8)	49 (6.2)	
Unknown	3 (0.4)	1 (0.1)	
Waist to hip ratio			
<0.8	589 (74.8)	573 (72.6)	0.33
≥0.8	198 (25.2)	216 (27.4)	
Unknown	4 (0.5)	2 (0.3)	
Smoking status			
Yes	66 (8.3)	62 (7.8)	0.78
No	725 (91.7)	724 (92.2)	
Diabetic status			
Yes	32 (4.0)	27 (3.4)	0.6
No	759 (96.0)	764 (96.6)	
Reported alcohol intake, g/day	11.7 ± 15.0	12.6 ± 15.4	0.25
Lifetime alcohol drinking pattern			
Non-consumers (0 g/day)	153 (19.5)	146 (18.5)	0.48
Light consumers (1–10 g/day)	334 (42.5)	321 (40.6)	
Drinkers (>10 g/day)	298 (38.0)	323 (40.9)	
Unknown	6 (0.8)	1 (0.1)	
Blood pressure status			
Normal	642 (81.8)	653 (83.0)	0.55
Hypertensive	143 (18.2)	133 (17.0)	
Unknown	6 (0.8)	5 (0.6)	
Previous use of oral contraceptives			
Yes	768 (97.7)	769 (97.8)	1
No	18 (2.3)	17 (2.1)	
Previous breastfeeding			
Yes	493 (63.9)	472 (61.1)	0.26
No	278^36^	301 (39.0)	
Unknown	6 (0.8)	5 (0.6)	
Menopause hormone therapy use at blood collection (post-menopausal)			
Yes	371 (46.9)	416 (52.6)	0.01
No	240 (30.3)	304 (24.8)	
Duration of use of menopause hormonal treatment, years (post-menopausal)	3.9 ± 4.5	4.4 ± 4.7	0.06

^a^Non-matching factors tested using a Chi-square test or Wilcoxon signed rank test.

### Association of untargeted profiles and individual metabolites with breast cancer risk

The processing of raw NMR spectra produced a matrix of 8500 chemical shift regions, with a median RSD of 6.9% across QCs (Supplementary Fig. S1). A median RSD in intensity of 6.9% among chemical shift regions (interquartile range: 1.3–18.7%) indicated that the analysis was reproducible and the data of high precision (Supplementary Fig. S2). BMI, diabetes status and collection centre accounted for most variability in raw metabolomics data (Supplementary Fig. S3). The PLS-DA model discriminated breast cancer cases from controls at blood collection modestly in premenopausal participants (ROC AUC = 0.61, 95% CI 0.49–0.73). For all other subgroups tested, an AUC of 0.5 was comfortably within 95% CIs, indicating predictions no better than random chance (Table 2).

**Table 2:** Receiver operating curve AUCs for prediction of breast cancer from untargeted NMR features.

Study group (cases and matched controls)	Number of cases and controls^a^	Median case time to diagnosis (years)	*k*-fold cross-validation procedure^b^	Receiver operating curve prediction accuracy for test set (AUC, 95% CI)^c^
All participants	1572	4.82	10-fold	0.51 (0.45–0.57)
Menopausal status at blood collection				
Pre-menopausal	354	5	5-fold, 5 times	0.61 (0.49–0.73)
Post-menopausal	1218	4.77	10-fold	0.53 (0.46–0.59)
Age at diagnosis				
<55 years	265	2.7	5-fold, 5 times	0.63 (0.49–0.77)
≥55 years	1307	5.48	10-fold	0.49 (0.43–0.55)
Fasting status at blood collection				
Yes	572	4.73	10-fold	0.52 (0.43–0.62)
No	1000	4.96	10-fold	0.53 (0.46–0.61)
Time from blood collection to cancer diagnosis				
<2 years	317	0.97	5-fold, 5 times	0.59 (0.46–0.72)
<5 years	752	7.67	10-fold	0.51 (0.43–0.59)

*AUC* area under curve.

CIs were estimated by DeLong’s method.

^a^Plasma samples of unknown waiting time to fractionation (*n* = 10) were excluded.

^b^Using a randomly selected 75% of the data.

^c^Using a randomly selected 25% of the data excluded from model training.

The 43 metabolites or biological indicators annotated according to 2D NMR chemical shift patterns comprised small alcohols and ketones, amino acids and other N-containing metabolites, organic acids, plasma proteins, cholines and three groups of fatty acids with distinct spectral characteristics. Ethanol was the only metabolite of direct exogenous origin, although no correlation was observed between plasma ethanol and reported alcohol intake on a continuous (*r* = –0.03) or categorical basis (*P* = 0.70 for quartiles of reported alcohol intake, Fig. 1b). Metabolites clustered strongly by correlation and concentrations of fatty acids were inversely correlated with those of other metabolites overall (Fig. 1c). Specific groups of metabolites, such as the branched-chain amino acids valine and leucine, were highly intercorrelated.

**Fig. 2:**
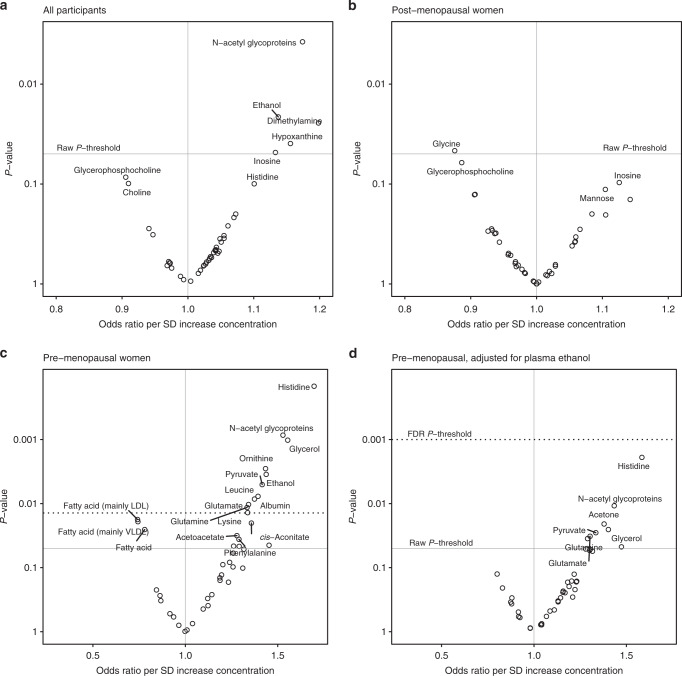
Odds ratios versus *P*-values (‘smile plots’) for metabolite univariate risk models by subgroup. **a** All study participants. **b** Post-menopausal women only. **c** Pre-menopausal women only. **d** Pre-menopausal women only and adjusted for plasma ethanol concentration. Raw *P*-values corresponding to FDR *P*-value thresholds could not be determined for (**a**, **b**). Models were adjusted for sample waiting time before plasma fractionation, BMI, diabetes status, waist-to-hip ratio, storage time, blood pressure, daily alcohol intake and duration of use of menopause hormonal treatments at blood collection. Five case–control pairs not matched on menopausal status were excluded from the analysis.

In the whole study, concentrations of N-acetyl glycoproteins (NAC), ethanol, hypoxanthine and dimethylamine, were positively associated with risk of breast cancer, although these associations were not significant after controlling for the false discovery rate (*P* = 0.162 and 0.351, respectively) (Fig. 2). In the premenopausal group however, breast cancer risk was associated with an increase in the concentrations of 10 metabolites after FDR adjustment for multiple testing. The strongest associations were observed for histidine (OR = 1.70 per SD increase in concentration, FDR-adjusted CI 1.19–2.41), glycerol (OR = 1.55 per SD increase, FDR-adjusted CI 1.11–2.18), NAC (OR = 1.53 per SD increase, FDR-adjusted CI 1.11–2.11) and ethanol (OR = 1.44 per SD increase, FDR-adjusted CI 1.05–1.97). Two of the fatty acid groups (mainly LDL and mainly VLDL) were borderline inversely associated with breast cancer risk (FDR-adjusted *P* = 0.062). Table 3 shows the results for those metabolites with raw *P*-values < 0.05 in at least one of the study groups. ORs and *P*-values for all metabolites are given overall and for pre- and postmenopausal subgroups in Supplementary Tables S3–S5, respectively.

**Table 3:** Odds ratios and confidence intervals for metabolites and breast cancer risk in the E3N cohort.

Plasma metabolite or metabolite group^a^	1D NMR ^1^H chemical shifts (ppm)	Study group	Scaled mean intensity (SD) in controls^b^	Scaled mean intensity (SD) in cases^b^	Odds ratio (95% CI) per SD increase in plasma concentration^c^	Odds ratio (FDR-adjusted CI) per SD increase^c^	Raw *P*-value	False discovery rate *P*-value
N-acetyl glycoproteins (Acetylation of proteins)	2.04, 2.07	All women	99.6 (8.4)	100.4 (8.3)	1.17 (1.05–1.31)	–	0.004	0.162
		Post-menopausal	99.4 (8.5)	99.7^8^	1.09 (0.96–1.24)	–	0.168	0.844
		Pre-menopausal	100.1 (8.1)	102.8^9^	1.53 (1.19–1.96)	1.53 (1.11–2.11)	0.001	0.015
		Pre-menopausal, adjusted ethanol	100.1 (8.1)	102.8^9^	1.43 (1.09–1.89)	–	0.011	0.194
Ethanol (Alcohol)	1.17	All women	99.9 (33.6)	100.1 (31.1)	1.14 (1.02–1.27)	–	0.021	0.351
		Post-menopausal	100.7 (37.9)	99.5 (30.1)	1.05 (0.93–1.20)	–	0.409	0.913
		Pre-menopausal	97.4 (8.1)	102.1 (34.4)	1.44 (1.13–1.83)	1.44 (1.05–1.97)	0.004	0.03
Histidine (Amino acid)	3.16, 3.23,	All women	99.4 (18.4)	100.6 (18.3)	1.10 (0.98–1.23)	–	0.1	0.535
	7.04, 7.73	Post-menopausal	99.6 (18.1)	99.5 (18.2)	0.97 (0.85–1.10)	–	0.615	0.973
		Pre-menopausal	98.6 (19.4)	104.2 (18.2)	1.70 (1.29–2.23)	1.70 (1.19–2.41)	<0.001	0.006
		Pre-menopausal, adjusted ethanol	98.6 (19.4)	104.2 (18.2)	1.58 (1.18–2.12)	–	0.002	0.08
Glycerol (Alcohol)	3.56, 3.65	All women	99.7 (13.7)	100.3 (13.8)	1.07 (0.96–1.19)	–	0.217	0.767
		Post-menopausal	99.3 (14.3)	98.9 (13.2)	0.97 (0.86–1.10)	–	0.649	0.973
		Pre-menopausal	101.3 (11.4)	105.2 (14.7)	1.55 (1.19–2.02)	1.55 (1.11, 2.18)	0.001	0.015
		Pre-menopausal, adjusted ethanol	101.3 (11.4)	105.2 (14.7)	1.40 (1.04–1.89)	–	0.025	0.194
Ornithine (Amino acid)	1.82	All women	99.8 (11.3)	100.2 (11.2)	1.07 (0.96–1.19)	–	0.2	0.767
		Post-menopausal	100.1 (11.6)	99.7 (11.4)	0.99 (0.87–1.12)	–	0.882	0.973
		Pre-menopausal	99 (10.2)	101.9 (10.5)	1.43 (1.13–1.82)	1.43 (1.06–1.95)	0.003	0.03
		Pre-menopausal, adjusted ethanol	99 (10.2)	101.9 (10.5)	1.30 (1.00–1.70)	–	0.052	0.194
Leucine (Amino acid)	1.70, 1.71, 3.72	All women	99.6 (9.5)	100.4 (10.3)	1.06 (0.96–1.18)	–	0.262	0.767
		Post-menopausal	99.6 (9.7)	100 (10.6)	1.00 (0.89–1.12)	–	0.959	0.973
		Pre-menopausal	99.6 (8.9)	101.7 (9.2)	1.37 (1.08–1.74)	1.37 (1.01–1.86)	0.009	0.046
		Pre-menopausal, adjusted ethanol	99.6 (8.9)	101.7 (9.2)	1.28 (1.00–1.64)	–	0.051	0.194
Albumin (Protein)	2.97	All women	99.9^13^	100.1^13^	1.04 (0.93–1.17)	–	0.468	0.767
		Post-menopausal	99.1 (13.2)	98.5 (12.7)	0.95 (0.83–1.08)	–	0.425	0.913
		Pre-menopausal	102.3 (12.2)	105.7 (12.4)	1.39 (1.09–1.78)	1.39 (1.02–1.91)	0.008	0.046
		Pre-menopausal, adjusted ethanol	102.3 (12.2)	105.7 (12.4)	1.30 (1.00–1.70)	–	0.054	0.194
Glutamine (Amino acid)	2.1, 2.13, 2.43, 2.46	All women	99.9 (12.6)	100.1 (12.7)	1.03 (0.92–1.15)	–	0.632	0.767
		Post-menopausal	99.7 (12.5)	99.1 (12.6)	0.94 (0.82–1.06)	–	0.314	0.913
		Pre-menopausal	100.5 (12.8)	103.7 (12.2)	1.33 (1.07–1.67)	1.33 (1.00–1.78)	0.012	0.049
		Pre-menopausal, adjusted ethanol	100.5 (12.8)	103.7 (12.2)	1.29 (1.02–1.63)	–	0.035	0.194
Glutamate (Amino acid)	2.04, 2.12, 2.34, 3.75	All women	99.8 (10.9)	100.2 (10.8)	1.03 (0.93–1.15)	–	0.557	0.767
		Post-menopausal	99.6 (10.9)	99.2 (10.8)	0.94 (0.83–1.07)	–	0.364	0.913
		Pre-menopausal	100.8 (10.9)	103.3 (10.1)	1.34 (1.07–1.68)	1.34 (1.00–1.79)	0.01	0.049
		Pre-menopausal, adjusted ethanol	100.8 (10.9)	103.3 (10.1)	1.30 (1.02–1.66)	–	0.032	0.194
Pyruvate (Organic acid)	2.36	All women	99.6 (13.6)	100.4 (14.6)	1.03 (0.92–1.15)	–	0.579	0.767
		Post-menopausal	99.3 (13.4)	98.9 (13.4)	0.94 (0.82–1.07)	–	0.331	0.913
		Pre-menopausal	100.7 (14.2)	105.4 (17.4)	1.42 (1.11–1.81)	1.42 (1.04–1.94)	0.005	0.036
		Pre-menopausal, adjusted ethanol	100.7 (14.2)	105.4 (17.4)	1.33 (1.03–1.73)	–	0.028	0.194

^a^Only metabolites whose FDR-adjusted *P*-values fell beneath the significance threshold of 0.05 in one of the study groups are tabulated.

^b^Where a value of 100 is attributed to the mean intensity in all participants.

^c^Models were adjusted for smoking status, diabetes status, BMI, waist to hip ratio, daily alcohol intake, duration of hormone treatment at blood collection, waiting time before plasma fractionation and biobank storage time.

In the premenopausal subgroup, the additional adjustment for plasma ethanol concentration caused most metabolite associations to be weakened (Table 3). Only histidine remained significant after multiple testing adjustment (OR = 1.58 per SD increase in concentration, FDR-adjusted CI 1.18–2.12). Acetone was the only metabolite to increase in strength of association but did not meet the *P*-value significance threshold. In other sensitivity analyses in this group, additional adjustment for lifetime alcohol drinking did not appreciably affect associations, and associations weakened or were no longer statistically significant when those cases who were diagnosed within 2 years of blood collection were excluded (Supplementary Table S6). No statistically significant associations were found in the ER+ subgroup.

The exclusion of non-fasting participants (around two-thirds overall) attenuated ORs in some cases, particularly in the postmenopausal subgroup. Most notably, total fatty acids became suggestively associated with breast cancer risk (OR = 1.32 per SD increase, 95% CI 1.03–1.67, FDR-adjusted *P* = 0.14). In the premenopausal subgroup, ORs for fasting participants remained unchanged or increased for histidine, NAC and ethanol, valine and leucine, although the association of glycerol with risk was diminished. *P*-values no longer met the FDR threshold due to the limited number of participants in these subgroups. ORs and *P*-values for all metabolites are given overall and for pre- and postmenopausal subgroups by fasting status in Supplementary Tables S7–S9, respectively. A comparison of overall and fasting data for pre- and postmenopausal subgroups is presented in Supplementary Fig. S4.

## Discussion

In this study, full NMR profiles of baseline plasma samples were able to discriminate between breast cancer cases and controls in premenopausal women only. Although no individual metabolite was significantly associated with breast cancer risk overall after FDR adjustment of *P*-values, 10 metabolites were positively associated with risk in the premenopausal subgroup, particularly histidine, glycerol, NAC and ethanol. Since no clear associations were found between metabolites and breast cancer risk in the larger postmenopausal subgroup, our study is the first to report differential metabolite associations with breast cancer by menopausal status.

Endogenous oestrogen production decreases substantially following the menopause and, since breast cancer is considered a hormone-dependent neoplasm,^6,21^ risk factors may vary according to menopausal status. However, previous studies on breast cancer and metabolomics have used predominantly or wholly postmenopausal participants. These studies usually reported inverse associations between disease risk and blood triglycerides, fatty acids or their derivatives,^8–11^ suggesting that the disease is preceded by a marked dyslipidaemia. Amino acids, conversely, were most commonly found to be positively associated with breast cancer risk, among them the branched-chain amino acids valine and leucine, as well as lysine, arginine, phenylalanine and glutamine.^12,13^ Multiple studies also found carnitine derivatives to be positively associated with the disease.^9,12,22^ Findings reported by menopausal status are scarce. In those studies that included premenopausal participants, case numbers were low and heterogeneity by menopausal status was either not detected for metabolites that were associated with breast cancer overall^9^ or not specifically examined.^11,13^

In our study, no metabolites met the *P*-value threshold for significance in the postmenopausal subgroup although some associations strengthened upon the exclusion of non-fasting participants and fatty acids in particular approached significance for a positive association with breast cancer risk. This finding appears contrary to those of previous studies, although our NMR-based method did not resolve individual fatty acids, which may elicit opposing bioactivities.^23^ In the premenopausal subgroup, several statistically significant associations were detected, the strongest of which was the N-acetylation of glycoproteins (NAC). NAC is involved in the activation of inflammatory pathways and is a robust indicator of systemic inflammation similar to C-reactive protein.^24^ Although not measured previously in prospective studies on breast cancer, it has been associated with an increased risk of all-cause and cancer-specific mortality.^25^ Also positively associated with risk was histidine, which has previously been implicated in breast and colorectal cancer as a necessary precursor of histamine, whose release is an early event in inflammatory responses and that is a regulator of cell proliferation.^26^ Other notable positive associations were observed for glutamate, which has previously been linked to insulin resistance^27^ and glycerol, which is perturbed in conditions of dyslipidaemia.^28^ The reduction is oestrogen levels post menopause and the associated physiological changes likely drive the differences in metabolite associations by menopausal status. A recent study suggested that menopause attenuates metabolism, particularly lipids and inflammatory biomarkers, independently of advancing age.^29^ Thus, our results on a relatively small proportion of the metabolome might reflect complex interactions between these factors and metabolic changes related to early carcinogenesis.

Alcohol intake is considered a risk factor for breast cancer in both pre- and postmenopausal women.^30^ In the full E3N cohort, associations were found for postmenopausal breast cancer risk only^31^ and the metabolomics subset under study was representative in this respect with a borderline positive association per 10 g of alcohol intake in postmenopausal women and no association in premenopausal women (data not shown). Two previous studies have linked circulating ethanol to breast cancer risk, predominantly in postmenopausal women: an NMR metabolomics study that found ethanol to be part of a profile predictive of disease development^7^ and a nested case–control study within the PLCO cohort that found ethanol glucuronide, a known biomarker of alcohol intake, to be associated with overall risk, as well as other metabolites originating from alcoholic drinks.^10^ Multiple mechanisms have been proposed to link ethanol and breast cancer risk. A portion of absorbed ethanol is converted by alcohol dehydrogenase to acetaldehyde, a carcinogen that promotes tumorigenesis by forming DNA adducts.^32^ Exposure to ethanol is known to disrupt endogenous metabolism. For example, blood lipids such as glycerophospholipids are perturbed by high alcohol intake,^33–35^ and amino acid profiles were perturbed by heavy alcohol intake in Japanese men.^36^ Levels of branched-chain amino acids, including leucine, were seen to increase in response to high wine intake.^37^ Metabolic changes due to alcohol intake are broad since regular ethanol exposure may disrupt the growth of gut microbiota and thus affect nutrient absorption, cause hepatocyte damage^38^ and react directly with endogenous metabolites.

We observed a strong association between plasma ethanol and premenopausal breast cancer risk only, in contrast to the associations for self-reported alcohol intake, and no detectable correlation between plasma ethanol and alcohol intake. Assuming our measurements of free plasma ethanol were representative of overall blood ethanol, this suggests that premenopausal cases consumed more alcohol than controls in the hours preceding blood collection even though no difference was observed in reported intakes between cases and controls. Also, the self-reporting of alcohol intake has been suggested to be subject to bias in observational studies.^39^ In addition, ethanol was strongly positively correlated with some amino acids and inversely correlated with overall fatty acids, and associations between plasma concentrations and breast cancer risk for most other metabolites disappeared or became weaker when plasma ethanol was included as an additional covariate in these models. Therefore, the possibility of residual confounding by alcohol intake should be considered.

The strengths of our study were its substantial size and capacity for subgroup analysis, and particularly the inclusion of more than 300 premenopausal breast cancer cases. Originating from a single country, detailed medical and lifestyle data were acquired and processed consistently. All samples were analysed on the same laboratory platform that has proven to be stable and robust, avoiding the need to account for inter-laboratory variability, and sample processing parameters were of negligible influence on metabolomics data. The study is also subject to certain limitations. Firstly, most participants had not fasted prior to blood collection and time since the last meal was not recorded. However, sensitivity analyses suggested little effect of fasting status upon the polar metabolites most strongly associated with premenopausal breast cancer, and the distinction between pre- and postmenopausal associations remained. Blood samples were only available at a single timepoint meaning that the reproducibility of metabolite measurements could not be assessed, although it is likely that most of these endogenous metabolites are biologically reproducible within a 2-year period.^40^ Also, NMR-based metabolomics does not allow the identification of metabolites to the same degree as mass spectrometry-based platforms, and fewer metabolites were included than in other recent studies, representing a small proportion of the metabolome only. The metabolite set was nonetheless diverse and included representatives of important pathways. Finally, associations for some metabolites weakened upon exclusion of diagnoses made within 2 years of baseline, suggesting the presence, to some extent, of reverse causation.

In summary, untargeted plasma NMR profiles at blood collection were modestly predictive of breast cancer in premenopausal women only. However, differential metabolite associations with breast cancer were found for pre- and postmenopausal women. The metabolites most associated with the disease in premenopausal women were correlated to a substantial extent with plasma ethanol, suggesting residual confounding by alcohol intake. Stratification by menopausal status and a more meticulous consideration of alcohol intake, either by measurement error correction or the use of biomarkers, is therefore needed in future studies of the disease.

## Disclaimer

Where authors are identified as personnel of the International Agency for Research on Cancer/World Health Organization, the authors alone are responsible for the views expressed in this article and they do not necessarily represent the decisions, policy or views of the International Agency for Research on Cancer/World Health Organization.

## Supplementary information

Supplementary material

## Data Availability

Data are available from the corresponding author upon reasonable request.
