# Supplementary material for: Investigation of circulating metabolites associated with breast cancer risk by untargeted metabolomics: a case–control study nested within the French E3N cohort
Source: Br J Cancer. 2021 Mar 15;124(10):1734–43. doi: 10.1038/s41416-021-01304-1 (PMC8110540; doi:10.1038/s41416-021-01304-1)
Supplement: Supplementary file 1 — Supplementary material [file 41416_2021_1304_MOESM1_ESM.docx]

**SUPPLEMENTARY INFORMATION**

Jobard et al*. Investigation of circulating metabolites associated with breast cancer risk by untargeted metabolomics: a case-control study nested within the French E3N cohort*

## Supplementary Methods

### Preparation of plasma samples

Plasma samples stored at –196°C in cryostraws were transferred to the *Institut des Sciences Analytiques* and stored at -80°C until analysis. Before NMR data acquisition, batches of blood samples were thawed at room temperature and processed daily as described by Beckonert et al (1). 200 µl of each plasma samples were diluted with 400 µl of a 0.9% saline solution (NaCl 0.9% wt/vol, D_2_O 10% vol/vol) in a microtube and centrifuged for 5 min at 4°C at 12,000 g. Finally, 550 µl of supernatant was transferred to 5 mm NMR tubes. Samples were kept at 4°C for no more than 24h until analysis.

To check the stability of NMR data, two quality control plasma samples (QC) were prepared in parallel with experimental samples. QCs, provided by the EFS, were aliquots of plasma collected in the presence of citrate from one healthy donor. A QC was placed at the beginning and end of each sample rack for one day of NMR throughput (around 40 samples) to evaluate variability over this period. A total of 112 QCs were therefore analysed, or 7% of all samples run.

### Nuclear magnetic resonance spectroscopy

NMR spectra were acquired on a Bruker Avance III spectrometer operating at 600.55 MHz (^1^H resonance frequency) equipped with automatic sample changer with cooling capacity and a 5 mm TCI triple resonance cryoprobe. The temperature was regulated at 310 K throughout. Automatic 3D shimming was performed once per day on the first QC plasma sample. Prior to NMR data acquisition, automatic tuning and matching, frequency locking on D_2_O and 1D shimming were performed for each sample. Standard ^1^H 1D NMR pulse sequences, NOESY and CPMG with water pre-saturation, were applied on each sample to obtain corresponding metabolic profiles. A total of 128 transient free induction decays (FID) were collected for each experiment into 32,690 points over a spectral width of 12019.23 Hz (20 ppm). For both sequences, the acquisition time was set to 1.36 s with a relaxation delay (d1) of 2 s. The 90° pulse sequence length (p1) was automatically calibrated for each sample at around 13 µs, at a power level (plw1) of 7.78 W. The NOESY mixing time was set to 100 ms. The CPMG spin-echo delay was 300 µs, for a total CPMG filter length of 80 ms allowing an efficient attenuation of the lipid NMR signals. All FIDs were multiplied by an exponential weighting function corresponding to a 0.3 Hz line broadening factor before Fourier transformation. Spectra were manually phased and corrected for the baseline, and referenced to the anomeric proton doublet signal of -glucose at 5.23 ppm. Additional two-dimensional NMR spectra (^1^H-^1^H J-resolved (Jres), ^1^H-^1^H TOCSY, and ^1^H-^13^C HSQC correlation spectra) were acquired from one case and one control sample to assign NMR signals observed in the ^1^H one-dimensional fingerprints to metabolites.

### Metabolite assignment

The measured ^1^H and ^13^C chemical shifts for detected compounds were compared to data available in the HMDB (2) and ChenomX NMR Suite (ChenomX Inc., Edmonton, Canada) databases. Figure S1 shows the mean CPMG spectrum with metabolite assignments. Details of the 56 annotated metabolites are given in Table S1.

### Data matrices for statistical analyses

One-dimensional spectral data were imported into AMIX software (Bruker, GmbH, Rheinstetten, Germany). They were reduced over the chemical range of 0.5-9 ppm to 8,500 bins (buckets), each 0.001 ppm wide, for integration of signal intensity. The spectral ranges corresponding to residual water signal (4.14-5.11 ppm), to anticoagulant citrate signal (2.50-2.56 and 2.63-2.68) and to contamination by polyethylene glycol suspected to be present in plastic cryo-straws (3.68-3.72 ppm) were set to zero and spectra were normalized to their total intensity. Spectral ranges for which small shifts were observed between spectra were realigned using the Icoshift algorithm (3) in MATLAB software (The MathWorks Inc., Natick, MA). Spectra were Pareto scaled i.e. each bucket was mean-centered and divided by the square root of its standard deviation (4).

For univariate analyses, the statistical recoupling of variables (SRV) method (5) was first applied to group the 8,500 NMR variables (7,384 non-zero variables) into 243 intelligent buckets, clusters of NMR variables that correspond to reconstructions of peak entities. Clusters of variables corresponding to different peaks of the same metabolite (based on the metabolite identification reported above) were then combined into a single variable by summing up the bins. This procedure resulted in a list of 43 combined clusters of variables, which corresponded to distinct metabolites or lipid classes and were retained for further univariate analyses.

Prior to statistical analyses, 28 of 1610 plasma samples in the study were excluded because of poor spectral quality as detected by NMR. Therefore, the final study group for the present analysis included 791 BC cases and 791 matched controls.

### References

1. Beckonert O., Keun H.C., Ebbels T.M.D., Bundy J.G., Holmes E., Lindon J.C.*,* et al. Metabolic profiling, metabolomic and metabonomic procedures for NMR spectroscopy of urine, plasma, serum and tissue extracts. Nat Protoc **2**, 2692-703 (2007).

2. Wishart D.S., Knox C., Guo A.C., Eisner R., Young N., Gautam B.*,* et al*.* HMDB: a knowledgebase for the human metabolome. Nucleic Acids Res **37**, D603-D610 (2009).

3. Savorani F., Tomasi G., Engelsen S.B. icoshift: A versatile tool for the rapid alignment of 1D NMR spectra. J Magn Reson **202**, 190-202 (2007).

4. van den Berg R.A., Hoefsloot H.C.J., Westerhuis J.A., Smilde A.K., van der Werf M..J. Centering, scaling, and transformations: improving the biological information content of metabolomics data. BMC Genomics **7** (2006).

5. Blaise B.J., Shintu L., Elena B., Emsley L., Dumas M.E., Toulhoat P. Statistical Recoupling Prior to Significance Testing in Nuclear Magnetic Resonance Based Metabonomics. Anal Chem **81,** 6242-51 (2009).

## Supplementary Tables and Figures

**Figure S1.** ^1^H NMR CPMG mean spectrum of E3N breast sub-cohort plasma samples acquired at 600MHz. All plasma samples were collected in citrated tubes.

##
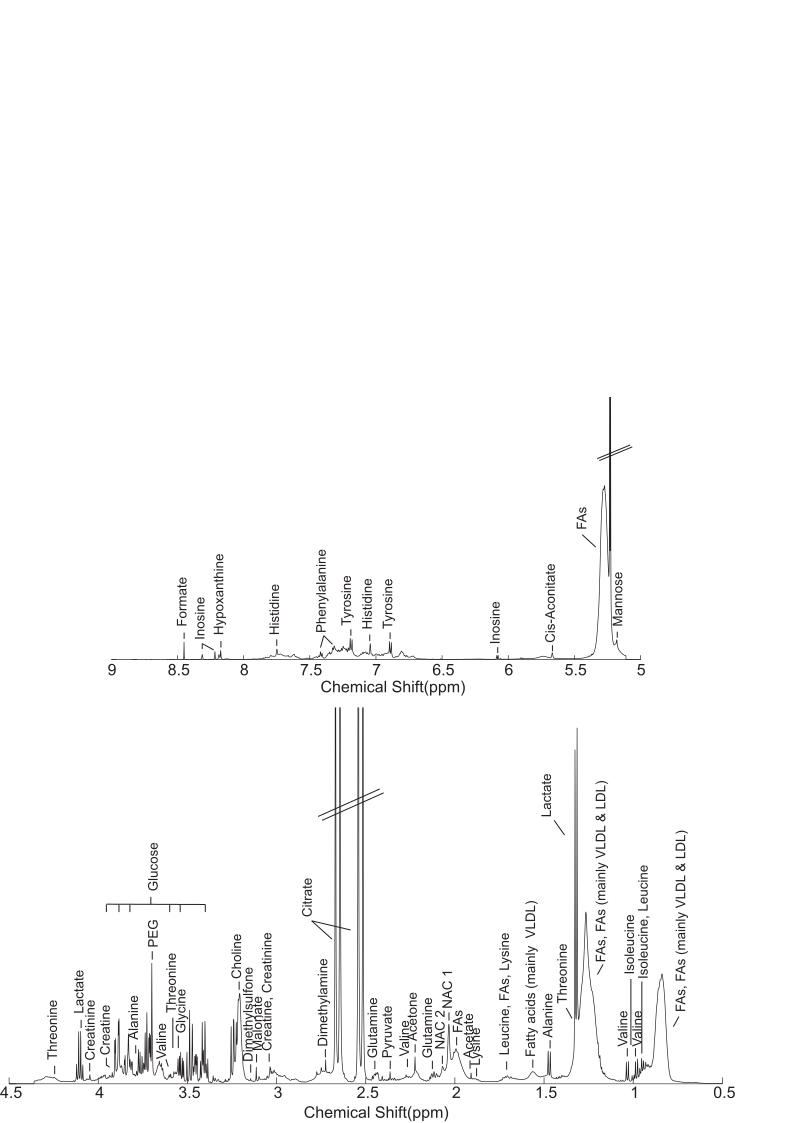


NAC: N-acetyl glycoproteins; FAs: Fatty acids.

**Table S1.** Metabolites identified from 1D and 2D NMR profiles of blood sera from participants of E3N breast sub-cohort.

| Metabolite | ^1^H ppm | ^13^C ppm | Multiplicity^a^ | Group | Observed |
| --- | --- | --- | --- | --- | --- |
| 3-hydroxybutyrate | 1.19 |  | d | ɣ-CH_3_ | CPMG, Jres |
|  | 2.3 |  | m |  | CPMG, Jres |
|  | 2.39 |  | m |  | CPMG, Jres |
|  | 4.14 |  | q |  | CPMG, Jres |
| Acetate | 1.91 | 26.1 | s | CH_3_ | CPMG, Jres, HSQC, TOCSY |
| Acetoacetate | 2.27 |  | s | CH_3_ | CPMG |
|  | 3.43 |  | s |  | CPMG |
| Acetone | 2.22 |  | s | CH_3_ | CPMG, Jres, TOCSY |
| Adipate | 1.5 | 30.4 | m |  | CPMG, HSQC |
| Albumin Lysyl | 2.97 | 42.1 | t | ε-CH_2_ | Jres, HSQC |
| Betaine | 3.26 | 56.2 | s | CH_3_ | CPMG, Jres, HSQC |
| Cholesterol | 0.66 | 14.4 | m | C18 (in HDL) | CPMG, HSQC, TOCSY |
|  | 0.72 | 25 | m | C18 (in LDL) | HSQC |
|  | 0.84 | 25.1 | m | C26 and C27 | CPMG, Jres, TOCSY, HSQC |
|  | 0.91 | 21.2 |  | C21 | Jres, HSQC |
| Choline | 3.19 | 56.7 | s | N(CH_3_)_3_ | CPMG, Jres, HSQC |
|  | 4.06 | 58 | s |  | HSQC |
| cis-Aconitate | 3.1 |  | d |  | CPMG, Jres |
|  | 5.66 |  | d |  | CPMG, Jres, TOCSY |
| Citrate | 2.53 | 48.2 | d | half CH_2_ | CPMG, Jres, HSQC, TOCSY |
|  | 2.65 | 48.2 | d | half CH_2_ | CPMG, Jres, HSQC, TOCSY |
| Creatine | 3.03 |  | s |  | CPMG, Jres |
|  | 3.92 |  | s | CH_2_ | CPMG, Jres |
| Creatinine | 3.04 |  | s |  | CPMG, Jres |
|  | 4.05 |  | s | CH_2_ | CPMG, Jres |
| D-Galactose | 5.26 | 95.2 | d |  | HSQC |
| D-Glucose (α) | 3.41 | 72.4 | t | H4 | CPMG, Jres, HSQC |
|  | 3.53 | 74.2 | dd | H2 | CPMG, Jres, TOCSY, HSQC |
|  | 3.71 | 75.7 | t | H3 | CPMG, Jres, HSQC |
|  | 3.72 | 68.7 | dd | half CH_2_C6 | CPMG, Jres, TOCSY, HSQC |
|  | 3.76 | 63.6 | q | half CH_2_C6 | CPMG, Jres, TOCSY, HSQC |
|  | 3.82 | 63.4/ 74.1 | m | H5 | CPMG, Jres, HSQC |
|  | 3.84 | 74.1 /63.5 | m | half CH_2_C6 | CPMG, Jres, HSQC |
|  | 5.23 | 94.9 | d | H1 | CPMG, Jres, TOCSY, HSQC |
| D-Glucose (β) | 3.24 | 77.1 | dd | H2 | CPMG, Jres, TOCSY, HSQC |
|  | 3.40 | 72.4 | t | H4 | CPMG, Jres, TOCSY, HSQC |
|  | 3.46 | 78.8 | m | H5 | CPMG, Jres, TOCSY, HSQC |
|  | 3.49 | 78.8 | t | H3 | CPMG, Jres, TOCSY, HSQC |
|  | 3.89 | 63.8 | q | half CH_2_C6 | CPMG, Jres, TOCSY, HSQC |
|  | 4.64 | 98.8 | d | H1 | CPMG, HSQC |
| D-Mannose | 5.18 | 96.5 | d |  | CPMG, Jres, TOCSY, HSQC |
| Dimethylamine | 2.72 |  | s |  | CPMG, Jres, TOCSY |
| Dimethylsulfone | 3.13 |  | s |  | CPMG, Jres |
| Ethanol | 1.17 |  | t |  | CPMG, Jres |
| Fatty acid | 1.21 |  | m | CH_3_CH_2_CH_2_ | CPMG, Jres, TOCSY |
|  | 1.24 | 25.1 | m | CH_3_CH_2_(CH_2_)n | CPMG, Jres, HSQC, TOCSY |
|  | 1.28 | 22.6 | m |  | CPMG, HSQC, TOCSY |
|  | 1.29 |  | m | CH_2_ | Jres |
|  | 1.69 | 29.1 | m | CH_2_CH_2_C=C | CPMG, Jres, TOCSY, HSQC |
|  | 1.99 | 29.7 | m | CH_2_C=C | Jres, TOCSY, HSQC |
|  | 2.19 | 36.4 | m | CH_2_CO | CPMG, TOCSY, HSQC |
|  | 2.23 | 36.4 | m | CH_2_CO | CPMG, TOCSY, HSQC |
|  | 2.69 |  | m | C=CCH_2_C=C | CPMG, TOCSY, HSQC |
|  | 2.72 | 28.1 | m | C=CCH_2_C=C | CPMG, HSQC |
|  | 5.25 |  | m | CH=CHCH_2_CH=CH | Jres |
|  | 5.27 |  | m |  | CPMG, Jres, TOCSY |
|  | 5.28 | 131.2 | m | (=CHCH2CH2) | CPMG, Jres, TOCSY |
|  | 5.29 | 124.6 | m | CH=CHCH_2_CH=CH | CPMG, Jres |
|  | 5.30 |  | m |  | CPMG, Jres, TOCSY |
|  | 5.32 |  | m | (=CHCH2CH2) | CPMG |
| Fatty acid (mainly LDL) | 0.86 | 16.5 | m | CH_3_(CH_2_)_n_ | Jres, TOCSY, HSQC |
|  | 1.26 | 32.6 | s | (CH_2_)n | CPMG, Jres, TOCSY, HSQC |
| Fatty acid (mainly VLDL) | 0.88 | 13.8 | t | CH_3_CH_2_CH_2_C= | Jres, HSQC |
|  | 1.26 | 24.2 | m | CH_2_CH_2_CH_2_CO | CPMG, TOCSY, HSQC |
|  | 1.29 |  | m | CH_2_CH_2_CH_2_CO | CPMG |
|  | 1.54 | 27.4 | m | CH_2_CH_2_CO | CPMG, TOCSY, HSQC |
| Formate | 8.45 |  | s | CH | CPMG, Jres, TOCSY |
| Glutamine | 2.1 |  | m |  | CPMG, Jres |
|  | 2.13 | 29.8 | m |  | CPMG, HSQC |
|  | 2.43 | 32.3 | m | half ɣ-CH_2_ | CPMG, Jres, HSQC |
|  | 2.46 |  | m |  | CPMG |
| Glycerol | 3.56 |  | q | half CH_2_ | CPMG, Jres |
|  | 3.65 | 65.6 | dd | half CH_2_ | CPMG, Jres, HSQC |
| Glycerol of backbone of PGLYs or TAGs | 4.06 | 64.5 | m | CHOCOR | CPMG, TOCSY, HSQC |
|  | 4.25 | 64.5 | m | CHOCOR | CPMG, TOCSY, HSQC |
|  | 5.2 | 71.6 | m | CHOCOR | CPMG, TOCSY, HSQC |
| Glycerophoshocholine | 3.21 | 56.8 | s |  | Jres, HSQC |
|  | 3.66 | 64.5 | m | NCH_2_ | CPMG, Jres, TOCSY, HSQC |
|  | 4.29 | 62.2 | m | OCH_2_ | NOESY, HSQC |
| Glycine | 3.55 | 44.4 | s | CH_2_ | CPMG, Jres, TOCSY, HSQC |
| Hypoxanthine | 8.17 | 148.4 | s |  | CPMG, Jres, TOCSY, HSQC |
|  | 8.18 | 144.9 | s |  | CPMG, TOCSY, HSQC |
| Inosine | 6.08 |  | d |  | CPMG, TOCSY |
|  | 8.22 |  | s |  | CPMG, Jres, TOCSY |
|  | 8.31 |  | s |  | CPMG, Jres, TOCSY |
| Isopropryl alcohol | 1.16 |  | d |  | Jres |
| Alanine | 1.47 | 19.08 | d | CH_3_ | CPMG, Jres, TOCSY, HSQC |
|  | 3.78 | 53.4 | q | α-CH | CPMG, Jres, TOCSY, HSQC |
| Aspartate | 2.67 | 39.4 | dd |  | CPMG, HSQC |
|  | 2.80 | 39.3 | dd |  | CPMG, HSQC |
|  | 2.89 | 55.2 | dd |  | HSQC |
| Glutamate | 2.04 | 29.8 | m |  | CPMG, HSQC |
|  | 2.12 | 29.8 | m |  | CPMG, Jres, HSQC |
|  | 2.34 | 36.2 | m |  | CPMG, Jres, HSQC |
|  | 3.75 | 57.5 | dd |  | CPMG, Jres, HSQC |
| Histidine | 3.16 | 30.7 | dd |  | HSQC |
|  | 3.23 | 30.7 | dd |  | HSQC |
|  | 7.04 | 120.3 | d | H4 | CPMG, HSQC |
|  | 7.73 | 121.1 | d |  | CPMG, Jres, HSQC, TOCSY |
| Isoleucine | 0.93 | 13.6 | t | δ-CH_3_ | CPMG, Jres, TOCSY, HSQC |
|  | 1.00 | 17.3 | d | δ-CH_3_ | CPMG, Jres, TOCSY, HSQC |
|  | 1.24 | 27 | m |  | HSQC |
|  | 1.45 | 27.2 | m |  | HSQC |
|  | 1.97 | 38.7 | m |  | HSQC |
|  | 3.66 | 62.4 | m |  | HSQC |
| Lactate | 1.32 | 22.3 | d | CH | CPMG, Jres, TOCSY, HSQC |
|  | 4.10 | 71.9 | q | CH_3_ | CPMG, Jres, TOCSY, HSQC |
| Lysine | 1.43 | 24.2 | m | ɣ-CH_2_ | CPMG, Jres, HSQC |
|  | 1.50 | 24.2 | m | ɣ-CH_2_ | CPMG, Jres, HSQC |
|  | 1.72 | 29.2 | m | δ-CH_2_ | CPMG, Jres, HSQC |
|  | 1.89 | 32.7 | m | β-CH_2_ | CPMG, Jres, HSQC |
|  | 3.02 | 42.1 | t |  | CPMG, Jres, HSQC |
|  | 3.75 | 57.4 | t |  | CPMG, Jres, HSQC |
| Methionine | 2.14 | 16.9 | s | S-CH_3_ | CPMG, Jres, TOCSY, HSQC |
|  | 2.63 | 31.6 | m |  | HSQC |
|  | 3.85 | 56.8 | m |  | HSQC |
| Ornithine | 1.82 | 25.6 | m |  | HSQC |
| Phenylalanine | 3.27 | 39.2 | dd |  | HSQC |
|  | 3.98 | 58.9 | q |  | Jres, HSQC |
|  | 7.32 | 132 | m | H2, H6 | CPMG, Jres, TOCSY, HSQC |
|  | 7.42 | 131.9 | m | H3, H5 | CPMG, Jres, TOCSY, HSQC |
| Proline | 2.00 | 26.6 | m |  | Jres, HSQC |
|  | 2.06 |  | m |  | Jres |
|  | 2.33 | 31.7 | m | half β-CH_2_ | Jres, HSQC |
|  | 3.33 | 48.9 | m |  | Jres, HSQC |
|  | 3.4 | 49 | m |  | HSQC |
|  | 4.12 | 64.1 | q |  | Jres, HSQC |
| Threonine | 1.31 | 22.3 | d | ɣ-CH_3_ | CPMG, Jres, HSQC |
|  | 3.60 | 63.3 | d | α-CH | CPMG, Jres, HSQC |
|  | 4.24 | 68.8 | m | β-CH_2_ | HSQC |
| Tryptophan | 3.47 | 29.3 | dd |  | HSQC |
|  | 7.19 | 122.3 | m |  | HSQC |
|  | 7.27 | 125 | m |  | HSQC |
|  | 7.53 | 114.7 | d |  | HSQC |
| Tyrosine | 3.05 | 38.4 | dd |  | HSQC |
|  | 3.18 | 38.3 | dd |  | HSQC |
|  | 3.93 | 58.9 | dd |  | HSQC |
|  | 6.89 | 118.8 | d | H2.H6 | CPMG, TOCSY, HSQC |
|  | 6.89 | 133.5 | s | H4 | CPMG, TOCSY, HSQC |
|  | 7.18 | 118.8 | s | H5 | CPMG, TOCSY, HSQC |
|  | 7.18 | 133.5 | d | H3, H5 | CPMG, TOCSY, HSQC |
| Valine | 0.98 | 19.4 | d | CH_3_ | CPMG, Jres, TOCSY, HSQC |
|  | 1.03 | 20.7 | d | CH_3_ | CPMG, Jres, TOCSY, HSQC |
|  | 2.26 | 31.9 | m | β-CH | CPMG, HSQC |
|  | 3.60 | 63.3 | d | α-CH | CPMG, Jres, HSQC |
| Lactose | 4.44 | 105.7 | d |  | HSQC |
| Leucine | 0.95 | 23.8 | d | δ-CH_3_ | CPMG, Jres, HSQC |
|  | 0.96 | 24.6 | d | δ-CH_3_ | CPMG, Jres, HSQC |
|  | 1.70 | 26.9/42.7 | m |  | CPMG, Jres, HSQC |
|  | 1.71 | 26.9/42.7 | m |  | CPMG, HSQC |
|  | 3.72 | 56.3 | m |  | HSQC |
| Malonate | 3.11 | 50.4 | s |  | CPMG, Jres, HSQC |
| Methanol | 3.36 |  | s |  | CPMG, Jres |
| Myo-inositol | 3.27 | 77.1 | t |  | CPMG, Jres, HSQC |
| NAC 1 | 2.04 | 25 | s | NHCOCH_3_ | CPMG, Jres, TOCSY, HSQC |
| NAC 2 | 2.07 | 25 | s | NHCOCH_3_ | CPMG, Jres, TOCSY, HSQC |
| PEG | 3.7 |  | s |  | CPMG, Jres |
| Pyruvate | 2.36 |  | s | CH_2_ | CPMG, Jres, TOCSY |
| Succinate | 2.4 |  | s |  | CPMG, Jres |

LDL: low density lipoproteins; VLDL: very low density lipoproteins; PGLYs; polyglycerides; TAGs: triacylglycerides; NAC, N-acetyl glycoproteins; PEG: polyethylene glycol.

^a^ Multiplicity of NMR signals: singlet (s), doublet (d), doublet of doublets (dd), triplet (t), quartet (q), multiplet (m).

**Table S2A.** Baseline characteristics of participants by menopausal status.

|  | Pre, controls (N=180) | Pre, cases (N=179) | Post, controls (N=611) | Post, cases (N=612) |
| --- | --- | --- | --- | --- |
| **Age at blood collection (years)** |  |  |  |  |
| Mean | 49.7 ± 2.3 | 49.7 ± 2.2 | 58.9 ± 5.9 | 58.9 ± 6.0 |
| **Follow-up time to cancer diagnosis** |  |  |  |  |
| 5 years or less | - | 89 (49.7) | - | 323 (52.8) |
| More than five years | - | 90 (50.3) | - | 289 (47.2) |
| **BMI** |  |  |  |  |
| Underweight or normal | 132 (73.7) | 140 (78.2) | 406 (66.7) | 424 (69.4) |
| Overweight | 39 (21.8) | 34 (19.0) | 165 (27.1) | 143 (23.4) |
| Obese | 8 (4.5) | 5 (2.8) | 38 (6.2) | 44 (7.2) |
| Unknown | 1 (0.6) | 0 (0.0) | 2 (0.3) | 1 (0.2) |
| **Waist to hip ratio** |  |  |  |  |
| < 0.8 | 145 (81.5) | 143 (79.9) | 444 (72.9) | 430 (70.5) |
| > 0.8 | 33 (18.5) | 36 (20.1) | 165 (27.1) | 180 (29.5) |
| Unknown | 2 (1.1) | 0 (0.0) | 2 (0.3) | 2 (0.3) |
| **Smoking status** |  |  |  |  |
| Yes | 21 (11.7) | 21 (11.7) | 45 (7.4) | 41 (6.7) |
| No | 159 (88.3) | 158 (88.3) | 566 (92.6) | 571 (93.3) |
| **Diabetic status** |  |  |  |  |
| Yes | 3 (1.7) | 7 (3.9) | 29 (4.7) | 20 (3.3) |
| No | 177 (98.3) | 172 (96.1) | 582 (95.3) | 592 (96.7) |
| **Lifetime alcohol drinking pattern** |  |  |  |  |
| Non-consumers (0 g/day) | 32 (17.8) | 27 (15.1) | 121 (20.0) | 119 (19.5) |
| Light consumers (1-10 g/day) | 73 (40.6) | 85 (47.5) | 261 (43.1) | 236 (38.6) |
| Drinkers (>10 g/day) | 75 (41.7) | 67 (37.4) | 223 (36.9) | 256 (41.9) |
| Unknown | 0 (0.0) | 0 (0.0) | 6 (1.0) | 1 (0.2) |
| **Alcohol intake (g/day)** |  |  |  |  |
| Mean (SD) | 12.9 ± 14.5 | 12.5 ± 15.1 | 11.4 ± 15.0 | 12.7 ± 15.6 |
| **Blood pressure** |  |  |  |  |
| Normal tension | 153 (85.5) | 157 (87.7) | 489 (80.7) | 496 (81.7) |
| Hypertension | 26 (14.5) | 22 (12.3) | 117 (19.3) | 111 (18.3) |
| Unknown | 1 (0.6) | 0 (0.0) | 5 (0.8) | 5 (0.8) |
| **Previous breastfeeding** |  |  |  |  |
| Yes | 110 (64.3) | 102 (59.0) | 383 (63.8) | 370 (61.7) |
| No | 61 (36) | 71 (41) | 217 (36) | 230 (38) |
| Unknown | 9 (5.0) | 6 (3.4) | 11 (1.8) | 12 (2.0) |
| **Previous oral contraceptive use** |  |  |  |  |
| Yes | 164 (91.1) | 164 (91.6) | 609 (99.7) | 610 (99.7) |
| No | 16 (8.9) | 15 (8.4) | 2 (0.3) | 2 (0.3) |
| **Menopause hormone therapy at blood collection** |  |  |  |  |
| Yes | - | - | 371 (60.7) | 416 (68.0) |
| No | - | - | 240 (39.3) | 196 (32.0) |
| Unknown | - | - | 0 (0.0) | 0 (0.0) |
| **Duration of use of menopause hormonal treatment at baseline** |  |  |  |  |
| Mean (SD) | - | - | 3.9 ± 4.5 | 4.4 ± 4.7 |
| **Fasting status** |  |  |  |  |
| Fasting | 63 (35.0) | 63 (35.2) | 224 (36.7) | 224 (36.6) |
| Non-fasting | 117 (65.0) | 116 (64.8) | 387 (63.3) | 388 (63.4) |
| **Biobank storage time (years)** |  |  |  |  |
| Mean (SD) | 14.0 ± 1.1 | 14.0 ± 1.2 | 13.5 ± 1.1 | 13.5 ± 1.1 |
|  |  |  |  |  |

**Table S2B.** Tumour characteristics of participants overall and by menopausal status.

| Characteristic | All cases (N=791) | Pre-menopausal cases (N=179) | Post-menopausal cases (N=611) |
| --- | --- | --- | --- |
|  |  | Mean ± SD or N (%) |  |
| **Age at diagnosis (years)** | 61.9 ± 7.0 | 55.1 ± 3.8 | 63.9 ± 6.5 |
| **Time from blood collection to diagnosis** |  |  |  |
| 5 years or less | 412 (52.1) | 89 (49.7) | 323 (52.8) |
| More than 5 years | 379 (47.9) | 90 (50.3) | 289 (47.2) |
| **Tumour behaviour** |  |  |  |
| In situ | 69 (8.7) | 16 (9.2) | 53 (9.1) |
| Invasive | 685 (86.6) | 157 (90.8) | 528 (90.9) |
| Unknown | 37 (4.7) | 6 (3.4) | 31 (5.1) |
| **Subtype** |  |  |  |
| Lobular | 110 (13.9) | 19 (11.0) | 91 (15.7) |
| Ductal | 360 (45.5) | 94 (54.3) | 266 (45.8) |
| Tubular | 25 (3.2) | 6 (3.5) | 19 (3.3) |
| Mixed | 11 (1.4) | 2 (1.2) | 9 (1.5) |
| Others | 248 (31.4) | 52 (30.1) | 196 (33.7) |
| Unknown | 37 (4.7) | 6 (3.4) | 31 (5.1) |
| **Oestrogen receptor** |  |  |  |
| Negative (ER-) | 100 (12.6) | 19 (13.1) | 81 (16.5) |
| Positive (ER+) | 536 (67.8) | 126 (86.9) | 410 (83.5) |
| Unknown | 155 (19.6) | 34 (19.0) | 121 (19.8) |
| **Progesterone receptor** |  |  |  |
| Negative | 193 (24.4) | 33 (24.3) | 160 (34.4) |
| Positive | 408 (51.6) | 103 (75.7) | 305 (65.6) |
| Unknown | 190 (24.0) | 43 (24.0) | 147 (24.0) |
| **Grade** |  |  |  |
| I | 221 (27.9) | 52 (36.1) | 169 (35.9) |
| II | 291 (36.8) | 65 (45.1) | 226 (48.0) |
| III | 103 (13.0) | 27 (18.8) | 76 (16.1) |
| Unknown | 176 (22.3) | 35 (19.6) | 141 (23.0) |
| **Stage** |  |  |  |
| 1 | 421 (53.2) | 95 (64.6) | 326 (64.2) |
| 2 | 195 (24.7) | 44 (29.9) | 151 (29.7) |
| 3 | 37 (4.7) | 8 (5.4) | 29 (5.7) |
| 4 | 2 (0.3) | 0 (0.0) | 2 (0.4) |
| Unknown | 136 (17.2) | 32 (17.9) | 104 (17.0) |

**Figure S2.** A) PCA score plot of the 1^H^ NMR CPMG profiles (8500 NMR chemical shift regions) of 1582 experimental plasma samples and 112 QC plasma samples acquired at 600 MHz over 10 weeks. The first and second principal components are shown. B) Distribution of relative standard deviations of the measured chemical shift regions in QCs.


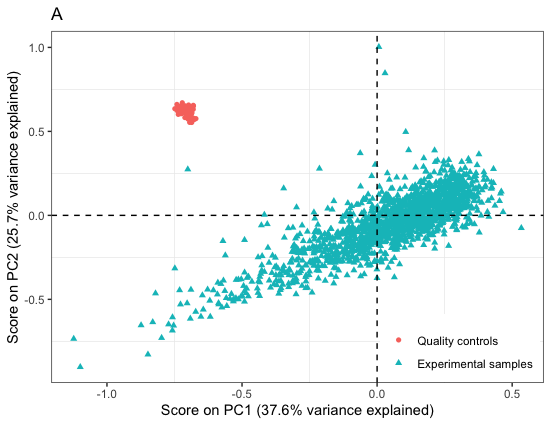


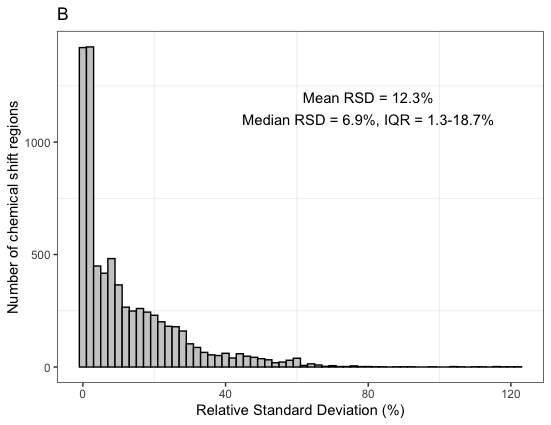


QC, quality control; RSD, relative standard deviation; IQR, interquartile range.

**Figure S3.** PC-PR2 analysis of 7384 NMR chemical shift regions before and after transformation of each variable to the residuals of a linear model of intensity on confounders.


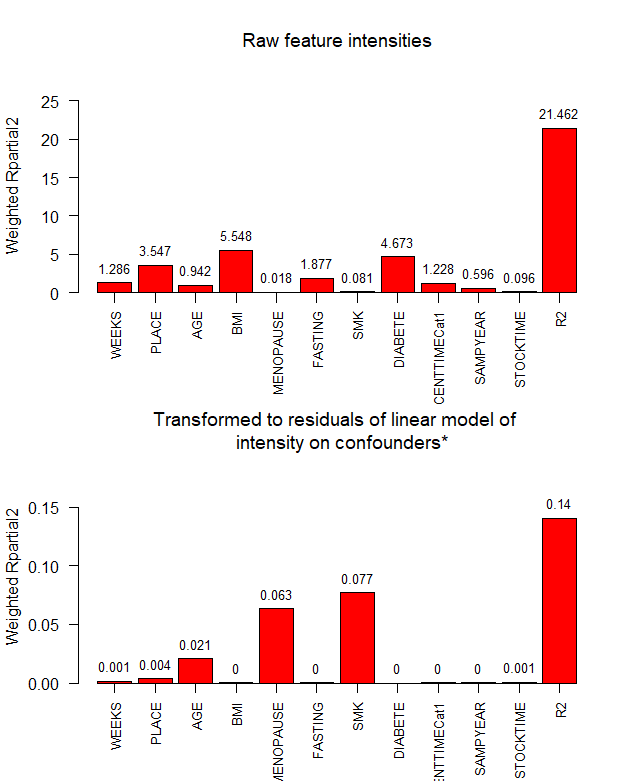


Adjusted for place of sample collection, year of collection, week of analysis, biobank storage time, age, BMI, diabetes status, fasting status, and sample waiting time before fractionation.

**Table S3.** Odds ratios and 95% CIs for risk of a breast cancer diagnosis per SD increase in metabolite concentration for all study participants.

| Metabolite or metabolite group | Description | Odds ratio^a^ | 95% confidence interval | Raw *P-*value | FDR-adjusted *P-*value |
| --- | --- | --- | --- | --- | --- |
| Ethanol | Alcohol | 1.14 | (1.02, 1.27) | 0.021 | 0.351 |
| Glycerol | Alcohol | 1.07 | (0.96, 1.19) | 0.217 | 0.767 |
| Methanol | Alcohol | 1.02 | (0.92, 1.14) | 0.653 | 0.767 |
| Creatine | Amine | 1.05 | (0.95, 1.16) | 0.354 | 0.767 |
| Creatinine | Amine | 1.03 | (0.93, 1.15) | 0.534 | 0.767 |
| Dimethylamine | Amine | 1.20 | (1.02, 1.4) | 0.025 | 0.351 |
| Glutamine | Amino acid | 1.03 | (0.92, 1.15) | 0.632 | 0.767 |
| Glycine | Amino acid | 0.94 | (0.84, 1.05) | 0.279 | 0.767 |
| Alanine | Amino acid | 0.99 | (0.89, 1.1) | 0.840 | 0.880 |
| Aspartate | Amino acid | 1.02 | (0.92, 1.14) | 0.653 | 0.767 |
| Glutamate | Amino acid | 1.03 | (0.93, 1.15) | 0.557 | 0.767 |
| Histidine | Amino acid | 1.10 | (0.98, 1.23) | 0.100 | 0.535 |
| Isoleucine | Amino acid | 1.00 | (0.9, 1.12) | 0.938 | 0.938 |
| Lysine | Amino acid | 1.04 | (0.94, 1.16) | 0.460 | 0.767 |
| Methionine | Amino acid | 1.04 | (0.93, 1.16) | 0.455 | 0.767 |
| Ornithine | Amino acid | 1.07 | (0.96, 1.19) | 0.200 | 0.767 |
| Phenylalanine | Amino acid | 1.05 | (0.95, 1.17) | 0.328 | 0.767 |
| Proline | Amino acid | 0.97 | (0.87, 1.08) | 0.619 | 0.767 |
| Tyrosine | Amino acid | 1.03 | (0.93, 1.14) | 0.604 | 0.767 |
| Valine | Amino acid | 1.02 | (0.92, 1.14) | 0.660 | 0.767 |
| Leucine | Amino acid | 1.06 | (0.96, 1.18) | 0.262 | 0.767 |
| Choline | Choline | 0.91 | (0.81, 1.02) | 0.099 | 0.535 |
| Glycerophosphocholine | Choline | 0.91 | (0.81, 1.01) | 0.085 | 0.535 |
| Fatty acid | Fatty acid | 0.95 | (0.85, 1.05) | 0.322 | 0.767 |
| Fatty acid (mainly LDL) | Fatty acid | 0.97 | (0.87, 1.08) | 0.597 | 0.767 |
| Fatty acid (mainly VLDL) | Fatty acid | 0.97 | (0.87, 1.09) | 0.615 | 0.767 |
| Glucose | Hexose sugar | 1.02 | (0.91, 1.14) | 0.784 | 0.842 |
| Mannose | Hexose sugar | 1.05 | (0.94, 1.17) | 0.381 | 0.767 |
| Acetone | Ketone | 1.04 | (0.93, 1.16) | 0.486 | 0.767 |
| Inosine | Nucleoside | 1.13 | (1, 1.28) | 0.048 | 0.416 |
| 3-Hydroxybutyrate | Organic acid | 1.02 | (0.92, 1.13) | 0.730 | 0.804 |
| Acetate | Organic acid | 1.04 | (0.92, 1.16) | 0.544 | 0.767 |
| Acetoacetate | Organic acid | 0.99 | (0.89, 1.11) | 0.906 | 0.928 |
| cis-Aconitate | Organic acid | 1.05 | (0.94, 1.18) | 0.350 | 0.767 |
| Formate | Organic acid | 1.05 | (0.92, 1.19) | 0.492 | 0.767 |
| Lactate | Organic acid | 0.97 | (0.84, 1.11) | 0.655 | 0.767 |
| Malonate | Organic acid | 1.05 | (0.92, 1.19) | 0.471 | 0.767 |
| Pyruvate | Organic acid | 1.03 | (0.92, 1.15) | 0.579 | 0.767 |
| Succinate | Organic acid | 0.98 | (0.86, 1.1) | 0.694 | 0.785 |
| Albumin | Protein | 1.04 | (0.93, 1.17) | 0.468 | 0.767 |
| N-acetyl-glycoproteins | Protein | 1.17 | (1.05, 1.31) | 0.004 | 0.162 |
| Hypoxanthine | Purine | 1.16 | (1.01, 1.33) | 0.039 | 0.416 |
| Cholesterol | Steroid | 1.04 | (0.94, 1.16) | 0.430 | 0.767 |

FDR, false discovery rate.

^a^ Models were adjusted for smoking status, diabetes status, BMI, waist to hip ratio, daily alcohol intake, duration of hormone treatment at diagnosis, sample waiting time before fractionation and biobank storage time.

**Table S4.** Odds ratios and 95% CIs for a breast cancer diagnosis per SD increase in metabolite concentration for participants who were pre-menopausal at blood collection.

| Metabolite or metabolite group | Description | Odds ratio^a^ | 95% confidence interval | Raw *P-*value | FDR-adjusted *P-*value |
| --- | --- | --- | --- | --- | --- |
| Ethanol | Alcohol | 1.44 | (1.13, 1.83) | 0.004 | 0.030 |
| Glycerol | Alcohol | 1.55 | (1.19, 2.02) | 0.001 | 0.015 |
| Methanol | Alcohol | 1.20 | (0.95, 1.51) | 0.130 | 0.207 |
| Creatine | Amine | 1.20 | (0.97, 1.49) | 0.090 | 0.161 |
| Creatinine | Amine | 0.96 | (0.74, 1.26) | 0.795 | 0.833 |
| Dimethylamine | Amine | 1.45 | (1.01, 2.09) | 0.045 | 0.100 |
| Glutamine | Amino acid | 1.33 | (1.07, 1.67) | 0.012 | 0.049 |
| Glycine | Amino acid | 1.12 | (0.9, 1.39) | 0.303 | 0.383 |
| Alanine | Amino acid | 1.01 | (0.79, 1.29) | 0.942 | 0.964 |
| Aspartate | Amino acid | 1.04 | (0.82, 1.31) | 0.747 | 0.803 |
| Glutamate | Amino acid | 1.34 | (1.07, 1.68) | 0.010 | 0.049 |
| Histidine | Amino acid | 1.70 | (1.29, 2.23) | 0.000 | 0.006 |
| Isoleucine | Amino acid | 1.00 | (0.79, 1.27) | 0.989 | 0.989 |
| Lysine | Amino acid | 1.34 | (1.06, 1.69) | 0.014 | 0.054 |
| Methionine | Amino acid | 1.26 | (0.99, 1.6) | 0.059 | 0.116 |
| Ornithine | Amino acid | 1.43 | (1.13, 1.82) | 0.003 | 0.030 |
| Phenylalanine | Amino acid | 1.29 | (1.02, 1.64) | 0.036 | 0.091 |
| Proline | Amino acid | 1.10 | (0.87, 1.39) | 0.443 | 0.515 |
| Tyrosine | Amino acid | 1.26 | (1, 1.58) | 0.046 | 0.100 |
| Valine | Amino acid | 1.19 | (0.94, 1.51) | 0.158 | 0.235 |
| Leucine | Amino acid | 1.37 | (1.08, 1.74) | 0.009 | 0.046 |
| Choline | Choline | 0.86 | (0.66, 1.12) | 0.273 | 0.356 |
| Glycerophosphocholine | Choline | 0.92 | (0.7, 1.2) | 0.524 | 0.593 |
| Fatty acid | Fatty acid | 0.78 | (0.63, 0.97) | 0.025 | 0.073 |
| Fatty acid (mainly LDL) | Fatty acid | 0.74 | (0.58, 0.95) | 0.018 | 0.062 |
| Fatty acid (mainly VLDL) | Fatty acid | 0.74 | (0.58, 0.95) | 0.019 | 0.062 |
| Glucose | Hexose sugar | 1.19 | (0.94, 1.49) | 0.144 | 0.222 |
| Mannose | Hexose sugar | 0.84 | (0.64, 1.11) | 0.223 | 0.309 |
| Acetone | Ketone | 1.29 | (1, 1.66) | 0.047 | 0.100 |
| Inosine | Nucleoside | 1.23 | (0.91, 1.66) | 0.169 | 0.242 |
| 3-Hydroxybutyrate | Organic acid | 1.24 | (0.97, 1.58) | 0.083 | 0.155 |
| Acetate | Organic acid | 1.14 | (0.9, 1.45) | 0.264 | 0.355 |
| Acetoacetate | Organic acid | 1.28 | (1.02, 1.6) | 0.032 | 0.085 |
| cis-Aconitate | Organic acid | 1.36 | (1.05, 1.76) | 0.020 | 0.062 |
| Formate | Organic acid | 1.12 | (0.86, 1.46) | 0.392 | 0.468 |
| Lactate | Organic acid | 0.87 | (0.65, 1.15) | 0.328 | 0.403 |
| Malonate | Organic acid | 1.32 | (1, 1.74) | 0.052 | 0.106 |
| Pyruvate | Organic acid | 1.42 | (1.11, 1.81) | 0.005 | 0.036 |
| Succinate | Organic acid | 1.26 | (0.96, 1.65) | 0.097 | 0.168 |
| Albumin | Protein | 1.39 | (1.09, 1.78) | 0.008 | 0.046 |
| N-acetyl-glycoproteins | Protein | 1.53 | (1.19, 1.96) | 0.001 | 0.015 |
| Hypoxanthine | Purine | 1.31 | (0.95, 1.81) | 0.102 | 0.168 |
| Cholesterol | Steroid | 0.94 | (0.74, 1.19) | 0.588 | 0.649 |

FDR, false discovery rate.

^a^ Models were adjusted for smoking status, diabetes status, BMI, waist to hip ratio, daily alcohol intake, duration of hormone treatment at diagnosis, sample waiting time before fractionation and biobank storage time.

**Table S5.** Odds ratios and 95% CIs for a breast cancer diagnosis per SD increase in metabolite concentration for participants who were post-menopausal at blood collection.

| Metabolite or metabolite group | Description | Odds ratio^a^ | 95% confidence interval | Raw *P-*value | FDR-adjusted *P-*value |
| --- | --- | --- | --- | --- | --- |
| Ethanol | Alcohol | 1.05 | (0.93, 1.2) | 0.409 | 0.913 |
| Glycerol | Alcohol | 0.97 | (0.86, 1.1) | 0.649 | 0.973 |
| Methanol | Alcohol | 0.98 | (0.87, 1.11) | 0.776 | 0.973 |
| Creatine | Amine | 1.01 | (0.9, 1.14) | 0.817 | 0.973 |
| Creatinine | Amine | 1.06 | (0.95, 1.2) | 0.303 | 0.913 |
| Dimethylamine | Amine | 1.14 | (0.96, 1.37) | 0.143 | 0.844 |
| Glutamine | Amino acid | 0.94 | (0.82, 1.06) | 0.314 | 0.913 |
| Glycine | Amino acid | 0.88 | (0.77, 1) | 0.052 | 0.844 |
| Alanine | Amino acid | 0.99 | (0.87, 1.12) | 0.868 | 0.973 |
| Aspartate | Amino acid | 1.02 | (0.91, 1.15) | 0.709 | 0.973 |
| Glutamate | Amino acid | 0.94 | (0.83, 1.07) | 0.364 | 0.913 |
| Histidine | Amino acid | 0.97 | (0.85, 1.1) | 0.615 | 0.973 |
| Isoleucine | Amino acid | 1.01 | (0.9, 1.14) | 0.847 | 0.973 |
| Lysine | Amino acid | 0.96 | (0.85, 1.09) | 0.569 | 0.973 |
| Methionine | Amino acid | 0.99 | (0.87, 1.12) | 0.882 | 0.973 |
| Ornithine | Amino acid | 0.99 | (0.87, 1.12) | 0.882 | 0.973 |
| Phenylalanine | Amino acid | 1.00 | (0.88, 1.13) | 0.973 | 0.973 |
| Proline | Amino acid | 0.94 | (0.83, 1.07) | 0.344 | 0.913 |
| Tyrosine | Amino acid | 0.97 | (0.86, 1.1) | 0.680 | 0.973 |
| Valine | Amino acid | 0.99 | (0.88, 1.12) | 0.892 | 0.973 |
| Leucine | Amino acid | 1.00 | (0.89, 1.12) | 0.959 | 0.973 |
| Choline | Choline | 0.91 | (0.8, 1.03) | 0.146 | 0.844 |
| Glycerophosphocholine | Choline | 0.89 | (0.79, 1.01) | 0.075 | 0.844 |
| Fatty acid | Fatty acid | 1.02 | (0.9, 1.16) | 0.737 | 0.973 |
| Fatty acid (mainly LDL) | Fatty acid | 1.05 | (0.93, 1.2) | 0.414 | 0.913 |
| Fatty acid (mainly VLDL) | Fatty acid | 1.06 | (0.93, 1.2) | 0.408 | 0.913 |
| Glucose | Hexose sugar | 0.95 | (0.84, 1.09) | 0.490 | 0.973 |
| Mannose | Hexose sugar | 1.10 | (0.97, 1.25) | 0.123 | 0.844 |
| Acetone | Ketone | 0.99 | (0.87, 1.12) | 0.857 | 0.973 |
| Inosine | Nucleoside | 1.12 | (0.97, 1.29) | 0.109 | 0.844 |
| 3-Hydroxybutyrate | Organic acid | 0.96 | (0.85, 1.09) | 0.539 | 0.973 |
| Acetate | Organic acid | 1.01 | (0.88, 1.15) | 0.921 | 0.973 |
| Acetoacetate | Organic acid | 0.91 | (0.8, 1.03) | 0.142 | 0.844 |
| cis-Aconitate | Organic acid | 1.00 | (0.88, 1.13) | 0.962 | 0.973 |
| Formate | Organic acid | 1.02 | (0.88, 1.17) | 0.832 | 0.973 |
| Lactate | Organic acid | 1.03 | (0.87, 1.21) | 0.753 | 0.973 |
| Malonate | Organic acid | 0.97 | (0.84, 1.12) | 0.659 | 0.973 |
| Pyruvate | Organic acid | 0.94 | (0.82, 1.07) | 0.331 | 0.913 |
| Succinate | Organic acid | 0.92 | (0.8, 1.06) | 0.255 | 0.913 |
| Albumin | Protein | 0.95 | (0.83, 1.08) | 0.425 | 0.913 |
| N-acetyl glycoproteins | Protein | 1.09 | (0.96, 1.24) | 0.168 | 0.844 |
| Hypoxanthine | Purine | 1.11 | (0.95, 1.3) | 0.177 | 0.844 |
| Cholesterol | Steroid | 1.06 | (0.94, 1.2) | 0.321 | 0.913 |

FDR, false discovery rate.

^a^ Models were adjusted for smoking status, diabetes status, BMI, waist to hip ratio, daily alcohol intake, duration of hormone treatment at diagnosis, sample waiting time before fractionation and biobank storage time.

**Table S6**. Additional sensitivity analyses for individual metabolites in the pre-menopausal subgroup.

| Plasma metabolite | Model^a^ | *N* | Odds ratio (95% CI) per SD increase in plasma concentration | Odds ratio (FDR-adjusted CI) per SD increase in plasma concentration | Raw *P*-value | FDR-adjusted *P-*value |
| --- | --- | --- | --- | --- | --- | --- |
| N-acetyl glycoproteins | Base co-variates only | 354 | 1.53 (1.19-1.96) | 1.53 (1.11-2.11) | 0.001 | 0.015 |
|  | Base + lifetime alcohol pattern | 354 | 1.55 (1.20-2.00) | 1.55 (1.20-2.00) | 0.001 | 0.012 |
|  | Base excluding cases diagnosed within 2 years of blood collection | 282 | 1.44 (1.09-1.90) | - | 0.01 | 0.153 |
|  | Fasting participants only | 126 | 1.94 (1.16-3.24) | - | 0.012 | 0.257 |
| Ethanol | Base co-variates only | 354 | 1.44 (1.13-1.83) | 1.44 (1.05-1.97) | 0.004 | 0.03 |
|  | Base + lifetime alcohol pattern | 354 | 1.44 (1.13-1.83) | 1.44 (1.05-1.96) | 0.004 | 0.031 |
|  | Base excluding cases diagnosed within 2 years of blood collection | 282 | 1.38 (1.06-1.80) | - | 0.02 | 0.162 |
|  | Fasting participants only | 126 | 1.92 (1.12-3.31) | - | 0.018 | 0.257 |
| Histidine | Base co-variates only | 354 | 1.70 (1.29-2.23) | 1.70 (1.19-2.41) | <0.001 | 0.006 |
|  | Base + lifetime alcohol pattern | 354 | 1.75 (1.32-2.31) | 1.75 (1.22-2.49) | <0.001 | 0.004 |
|  | Base excluding cases diagnosed within 2 years of blood collection | 282 | 1.61 (1.19-2.18) | - | 0.002 | 0.077 |
|  | Fasting participants only |  | 1.90 (1.15-3.15) |  | 0.012 | 0.257 |
| Glycerol | Base co-variates only | 354 | 1.55 (1.19-2.02) | 1.55 (1.11-2.18) | 0.001 | 0.015 |
|  | Base + lifetime alcohol pattern | 354 | 1.57 (1.20-2.04) | 1.57 (1.12-2.19) | 0.001 | 0.012 |
|  | Base excluding cases diagnosed within 2 years of blood collection | 282 | 1.51 (1.13-2.03) | - | 0.006 | 0.128 |
|  | Fasting participants only | 126 | 1.39 (0.90-2.15) | - | 0.14 | 0.333 |
| Ornithine | Base co-variates only | 354 | 1.43 (1.13-1.82) | 1.43 (1.06-1.95) | 0.003 | 0.03 |
|  | Base + lifetime alcohol pattern | 354 | 1.46 (1.15-1.86) | 1.46 (1.07-1.99) | 0.002 | 0.023 |
|  | Base excluding cases diagnosed within 2 years of blood collection | 282 | 1.30 (1.01-1.68) | - | 0.044 | 0.234 |
|  | Fasting participants only | 126 | 1.39 (0.93-2.07) | - | 0.112 | 0.329 |
| Leucine | Base co-variates only | 354 | 1.37 (1.08-1.74) | 1.37 (1.01-1.86) | 0.009 | 0.046 |
|  | Base + lifetime alcohol pattern | 354 | 1.38 (1.09-1.74) | 1.38 (1.02-1.86) | 0.008 | 0.044 |
|  | Base excluding cases diagnosed within 2 years of blood collection | 282 | 1.30 (0.99-1.69) | - | 0.055 | 0.235 |
|  | Fasting participants only | 126 | 1.05 (0.86-1.29) | - | 0.601 | 0.749 |
| Albumin | Base co-variates only | 354 | 1.39 (1.09-1.78) | 1.39 (1.02-1.91) | 0.008 | 0.046 |
|  | Base + lifetime alcohol pattern | 354 | 1.40 (1.09-1.78) | 1.40 (1.02-1.90) | 0.007 | 0.044 |
|  | Base excluding cases diagnosed within 2 years of blood collection | 282 | 1.35 (1.03-1.76) | - | 0.029 | 0.234 |
|  | Fasting participants only | 126 | 1.30 (0.82-2.05) | - | 0.26 | 0.431 |
| Glutamine | Base co-variates only | 354 | 1.33 (1.07-1.67) | 1.33 (1.00-1.78) | 0.012 | 0.049 |
|  | Base + lifetime alcohol pattern | 354 | 1.34 (1.07-1.68) | 1.34 (1.01-1.78) | 0.011 | 0.046 |
|  | Base excluding cases diagnosed within 2 years of blood collection | 282 | 1.21 (0.95-1.55) | - | 0.123 | 0.302 |
|  | Fasting participants only | 126 | 1.38 (0.88-2.14) | - | 0.158 | 0.338 |
| Glutamate | Base co-variates only | 354 | 1.34 (1.07-1.68) | 1.34 (1.00-1.79) | 0.01 | 0.049 |
|  | Base + lifetime alcohol pattern | 354 | 1.35 (1.07-1.69) | 1.35 (1.01-1.80) | 0.01 | 0.046 |
|  | Base excluding cases diagnosed within 2 years of blood collection | 282 | 1.23 (0.96-1.56) | - | 0.01 | 0.302 |
|  | Fasting participants only | 126 | 1.46 (0.92-2.32) | - | 0.11 | 0.329 |
| Pyruvate | Base co-variates only | 354 | 1.42 (1.11-1.81) | 1.42 (1.04-1.94) | 0.005 | 0.036 |
|  | Base + lifetime alcohol pattern | 354 | 1.42 (1.11-1.81) | 1.42 (1.04-1.93) | 0.005 | 0.034 |
|  | Base excluding cases diagnosed within 2 years of blood collection | 282 | 1.32 (1.01-1.73) | - | 0.04 | 0.234 |
|  | Fasting participants only | 126 | 1.44 (0.89-2.32) | - | 0.1361 | 0.333 |

FDR, false discovery rate.

Only metabolites whose FDR adjusted *P*-values fell under the significance threshold of 0.05 in the base model are tabulated.

^a^ Base model adjusted for smoking status, diabetes status, BMI, waist to hip ratio, daily alcohol intake, waiting time before plasma fractionation and biobank storage time.

**Table S7.** Odds ratios and 95% CIs for a breast cancer diagnosis per SD increase in metabolite concentration for fasting participants only.

| Metabolite or metabolite group | Description | Odds ratio^a^ | 95% confidence interval | Raw *P-*value | FDR-adjusted *P-*value |
| --- | --- | --- | --- | --- | --- |
| Ethanol | Alcohol | 1.04 | (0.86-1.26) | 0.6966 | 0.758 |
| Glycerol | Alcohol | 0.9 | (0.74-1.1) | 0.325 | 0.636 |
| Methanol | Alcohol | 0.95 | (0.79-1.15) | 0.5898 | 0.749 |
| Creatine | Amine | 0.92 | (0.78-1.1) | 0.3784 | 0.636 |
| Creatinine | Amine | 1.15 | (0.97-1.38) | 0.1133 | 0.518 |
| Dimethylamine | Amine | 0.95 | (0.73-1.24) | 0.7048 | 0.758 |
| Glutamine | Amino acid | 0.91 | (0.73-1.12) | 0.3682 | 0.636 |
| Glycine | Amino acid | 0.92 | (0.76-1.12) | 0.4121 | 0.636 |
| Alanine | Amino acid | 0.85 | (0.69-1.04) | 0.1184 | 0.518 |
| Aspartate | Amino acid | 0.99 | (0.83-1.18) | 0.9047 | 0.905 |
| Glutamate | Amino acid | 0.89 | (0.72-1.11) | 0.3063 | 0.636 |
| Histidine | Amino acid | 1.13 | (0.92-1.39) | 0.2317 | 0.554 |
| Isoleucine | Amino acid | 0.93 | (0.72-1.21) | 0.6067 | 0.749 |
| Lysine | Amino acid | 0.85 | (0.7-1.04) | 0.1189 | 0.518 |
| Methionine | Amino acid | 0.92 | (0.75-1.12) | 0.4054 | 0.636 |
| Ornithine | Amino acid | 0.91 | (0.74-1.11) | 0.353 | 0.636 |
| Phenylalanine | Amino acid | 0.89 | (0.74-1.07) | 0.2234 | 0.554 |
| Proline | Amino acid | 0.85 | (0.7-1.04) | 0.1205 | 0.518 |
| Tyrosine | Amino acid | 0.92 | (0.76-1.12) | 0.4143 | 0.636 |
| Valine | Amino acid | 1.04 | (0.85-1.27) | 0.6938 | 0.758 |
| Leucine | Amino acid | 1.05 | (0.86-1.29) | 0.6094 | 0.749 |
| Choline | Choline | 0.83 | (0.69-1.01) | 0.0598 | 0.518 |
| Glycerophosphocholine | Choline | 0.79 | (0.65-0.96) | 0.0179 | 0.518 |
| Fatty acid | Fatty acid | 1.14 | (0.94-1.38) | 0.1737 | 0.534 |
| Fatty acid (mainly LDL) | Fatty acid | 1.21 | (0.99-1.47) | 0.0683 | 0.518 |
| Fatty acid (mainly VLDL) | Fatty acid | 1.21 | (0.99-1.48) | 0.0657 | 0.518 |
| Glucose | Hexose sugar | 0.93 | (0.76-1.16) | 0.5287 | 0.733 |
| Mannose | Hexose sugar | 1.19 | (0.99-1.44) | 0.0668 | 0.518 |
| Acetone | Ketone | 1.04 | (0.86-1.26) | 0.6729 | 0.758 |
| Inosine | Nucleoside | 1.1 | (0.89-1.35) | 0.3691 | 0.636 |
| 3-Hydroxybutyrate | Organic acid | 0.85 | (0.68-1.07) | 0.172 | 0.534 |
| Acetate | Organic acid | 0.84 | (0.68-1.04) | 0.1142 | 0.518 |
| Acetoacetate | Organic acid | 0.86 | (0.69-1.05) | 0.1412 | 0.534 |
| cis-Aconitate | Organic acid | 0.93 | (0.76-1.14) | 0.48 | 0.688 |
| Formate | Organic acid | 0.87 | (0.7-1.07) | 0.1865 | 0.535 |
| Lactate | Organic acid | 0.95 | (0.75-1.2) | 0.6593 | 0.758 |
| Malonate | Organic acid | 0.86 | (0.68-1.09) | 0.2257 | 0.554 |
| Pyruvate | Organic acid | 0.91 | (0.74-1.11) | 0.3581 | 0.636 |
| Succinate | Organic acid | 0.98 | (0.79-1.22) | 0.8418 | 0.862 |
| Albumin | Protein | 0.86 | (0.7-1.06) | 0.1615 | 0.534 |
| N-acetyl glycoproteins | Protein | 1.08 | (0.88-1.31) | 0.4568 | 0.677 |
| Hypoxanthine | Purine | 1.08 | (0.84-1.39) | 0.5455 | 0.733 |
| Cholesterol | Steroid | 1.03 | (0.86-1.22) | 0.7672 | 0.805 |

FDR, false discovery rate.

^a^ Models were adjusted for smoking status, diabetes status, BMI, waist to hip ratio, daily alcohol intake, duration of hormone treatment at diagnosis, sample waiting time before fractionation and biobank storage time.

**Table S8.** Odds ratios and 95% CIs for a breast cancer diagnosis per SD increase in metabolite concentration for fasting pre-menopausal participants only.

| Metabolite or metabolite group | Description | Odds ratio^a^ | 95% confidence interval | Raw *P-*value | FDR-adjusted *P-*value |
| --- | --- | --- | --- | --- | --- |
| Ethanol | Alcohol | 1.92 | (1.12-3.31) | 0.0179 | 0.257 |
| Glycerol | Alcohol | 1.39 | (0.9-2.15) | 0.1393 | 0.333 |
| Methanol | Alcohol | 1.32 | (0.89-1.96) | 0.1728 | 0.339 |
| Creatine | Amine | 1.36 | (0.92-2) | 0.1239 | 0.333 |
| Creatinine | Amine | 1.23 | (0.79-1.91) | 0.3658 | 0.562 |
| Dimethylamine | Amine | 0.91 | (0.45-1.8) | 0.7774 | 0.857 |
| Glutamine | Amino acid | 1.38 | (0.88-2.14) | 0.1573 | 0.338 |
| Glycine | Amino acid | 1.14 | (0.77-1.68) | 0.5146 | 0.681 |
| Alanine | Amino acid | 0.61 | (0.36-1.05) | 0.0736 | 0.32 |
| Aspartate | Amino acid | 0.91 | (0.62-1.34) | 0.6363 | 0.782 |
| Glutamate | Amino acid | 1.46 | (0.92-2.32) | 0.1102 | 0.329 |
| Histidine | Amino acid | 1.9 | (1.15-3.15) | 0.0124 | 0.257 |
| Isoleucine | Amino acid | 1.01 | (0.59-1.72) | 0.9755 | 0.976 |
| Lysine | Amino acid | 1.36 | (0.87-2.13) | 0.1734 | 0.339 |
| Methionine | Amino acid | 1.48 | (0.87-2.5) | 0.1476 | 0.334 |
| Ornithine | Amino acid | 1.39 | (0.93-2.07) | 0.1119 | 0.329 |
| Phenylalanine | Amino acid | 1.31 | (0.86-2) | 0.2024 | 0.352 |
| Proline | Amino acid | 0.94 | (0.63-1.4) | 0.7454 | 0.843 |
| Tyrosine | Amino acid | 1.39 | (0.92-2.09) | 0.1147 | 0.329 |
| Valine | Amino acid | 1.61 | (0.99-2.61) | 0.0548 | 0.32 |
| Leucine | Amino acid | 1.83 | (1.08-3.11) | 0.0245 | 0.263 |
| Choline | Choline | 0.61 | (0.37-1) | 0.0497 | 0.32 |
| Glycerophosphocholine | Choline | 0.66 | (0.4-1.07) | 0.0929 | 0.329 |
| Fatty acid | Fatty acid | 0.77 | (0.51-1.15) | 0.2044 | 0.352 |
| Fatty acid (mainly LDL) | Fatty acid | 0.84 | (0.55-1.28) | 0.4137 | 0.613 |
| Fatty acid (mainly VLDL) | Fatty acid | 0.84 | (0.55-1.3) | 0.4365 | 0.626 |
| Glucose | Hexose sugar | 1.25 | (0.8-1.94) | 0.3247 | 0.517 |
| Mannose | Hexose sugar | 0.92 | (0.57-1.47) | 0.7204 | 0.837 |
| Acetone | Ketone | 1.4 | (0.84-2.33) | 0.1992 | 0.352 |
| Inosine | Nucleoside | 1.04 | (0.63-1.74) | 0.87 | 0.935 |
| 3-Hydroxybutyrate | Organic acid | 1.63 | (0.98-2.7) | 0.0573 | 0.32 |
| Acetate | Organic acid | 1.14 | (0.76-1.71) | 0.5225 | 0.681 |
| Acetoacetate | Organic acid | 1.44 | (0.93-2.21) | 0.0988 | 0.329 |
| cis-Aconitate | Organic acid | 1.13 | (0.72-1.76) | 0.5905 | 0.747 |
| Formate | Organic acid | 1.09 | (0.71-1.68) | 0.6873 | 0.821 |
| Lactate | Organic acid | 0.98 | (0.58-1.65) | 0.9481 | 0.971 |
| Malonate | Organic acid | 0.98 | (0.59-1.63) | 0.938 | 0.971 |
| Pyruvate | Organic acid | 1.44 | (0.89-2.32) | 0.1361 | 0.333 |
| Succinate | Organic acid | 1.6 | (0.96-2.65) | 0.0715 | 0.32 |
| Albumin | Protein | 1.3 | (0.82-2.05) | 0.2606 | 0.431 |
| N-acetyl glycoproteins | Protein | 1.94 | (1.16-3.24) | 0.0115 | 0.257 |
| Hypoxanthine | Purine | 1.75 | (0.95-3.25) | 0.0745 | 0.32 |
| Cholesterol | Steroid | 0.86 | (0.56-1.31) | 0.4815 | 0.668 |

FDR, false discovery rate.

^a^ Models were adjusted for smoking status, diabetes status, BMI, waist to hip ratio, daily alcohol intake, sample waiting time before fractionation and biobank storage time.

**Table S9.** Odds ratios and 95% CIs for a breast cancer diagnosis per SD increase in metabolite concentration for fasting post-menopausal participants only.

| Metabolite or metabolite group | Description | Odds ratio^a^ | 95% confidence interval | Raw *P-*value | FDR-adjusted *P-*value |
| --- | --- | --- | --- | --- | --- |
| Ethanol | Alcohol | 0.93 | (0.74-1.15) | 0.489 | 0.657 |
| Glycerol | Alcohol | 0.79 | (0.62-1.02) | 0.0678 | 0.182 |
| Methanol | Alcohol | 0.85 | (0.68-1.07) | 0.17 | 0.292 |
| Creatine | Amine | 0.85 | (0.69-1.04) | 0.1203 | 0.246 |
| Creatinine | Amine | 1.19 | (0.96-1.46) | 0.1064 | 0.246 |
| Dimethylamine | Amine | 0.97 | (0.72-1.3) | 0.8169 | 0.892 |
| Glutamine | Amino acid | 0.77 | (0.59-1) | 0.0485 | 0.149 |
| Glycine | Amino acid | 0.83 | (0.66-1.04) | 0.1102 | 0.246 |
| Alanine | Amino acid | 0.9 | (0.71-1.13) | 0.3522 | 0.505 |
| Aspartate | Amino acid | 1.04 | (0.85-1.27) | 0.6972 | 0.826 |
| Glutamate | Amino acid | 0.75 | (0.58-0.97) | 0.0301 | 0.143 |
| Histidine | Amino acid | 0.95 | (0.74-1.21) | 0.6824 | 0.826 |
| Isoleucine | Amino acid | 0.93 | (0.69-1.26) | 0.6396 | 0.809 |
| Lysine | Amino acid | 0.74 | (0.57-0.94) | 0.0144 | 0.143 |
| Methionine | Amino acid | 0.84 | (0.67-1.06) | 0.1511 | 0.292 |
| Ornithine | Amino acid | 0.76 | (0.59-0.98) | 0.0333 | 0.143 |
| Phenylalanine | Amino acid | 0.79 | (0.63-1) | 0.048 | 0.149 |
| Proline | Amino acid | 0.82 | (0.65-1.05) | 0.1164 | 0.246 |
| Tyrosine | Amino acid | 0.8 | (0.63-1.02) | 0.0669 | 0.182 |
| Valine | Amino acid | 0.96 | (0.76-1.21) | 0.7105 | 0.826 |
| Leucine | Amino acid | 0.97 | (0.77-1.23) | 0.8301 | 0.892 |
| Choline | Choline | 0.86 | (0.69-1.08) | 0.1911 | 0.316 |
| Glycerophosphocholine | Choline | 0.79 | (0.63-1) | 0.0469 | 0.149 |
| Fatty acid | Fatty acid | 1.32 | (1.03-1.67) | 0.0253 | 0.143 |
| Fatty acid (mainly LDL) | Fatty acid | 1.37 | (1.07-1.76) | 0.013 | 0.143 |
| Fatty acid (mainly VLDL) | Fatty acid | 1.37 | (1.07-1.76) | 0.0131 | 0.143 |
| Glucose | Hexose sugar | 0.85 | (0.66-1.1) | 0.2126 | 0.339 |
| Mannose | Hexose sugar | 1.29 | (1.03-1.6) | 0.0237 | 0.143 |
| Acetone | Ketone | 1.01 | (0.82-1.25) | 0.933 | 0.955 |
| Inosine | Nucleoside | 1.16 | (0.91-1.47) | 0.2307 | 0.354 |
| 3-Hydroxybutyrate | Organic acid | 0.73 | (0.55-0.97) | 0.0296 | 0.143 |
| Acetate | Organic acid | 0.76 | (0.58-0.99) | 0.0435 | 0.149 |
| Acetoacetate | Organic acid | 0.72 | (0.55-0.94) | 0.0146 | 0.143 |
| cis-Aconitate | Organic acid | 0.89 | (0.71-1.13) | 0.3466 | 0.505 |
| Formate | Organic acid | 0.84 | (0.65-1.08) | 0.1627 | 0.292 |
| Lactate | Organic acid | 0.97 | (0.74-1.27) | 0.809 | 0.892 |
| Malonate | Organic acid | 0.82 | (0.62-1.08) | 0.1616 | 0.292 |
| Pyruvate | Organic acid | 0.8 | (0.63-1.02) | 0.0738 | 0.187 |
| Succinate | Organic acid | 0.89 | (0.69-1.16) | 0.3825 | 0.531 |
| Albumin | Protein | 0.75 | (0.58-0.97) | 0.0286 | 0.143 |
| N-acetyl glycoproteins | Protein | 0.94 | (0.74-1.19) | 0.6265 | 0.809 |
| Hypoxanthine | Purine | 1 | (0.75-1.32) | 0.9823 | 0.982 |
| Cholesterol | Steroid | 1.02 | (0.84-1.24) | 0.856 | 0.898 |

FDR, false discovery rate.

^a^ Models were adjusted for smoking status, diabetes status, BMI, waist to hip ratio, daily alcohol intake, sample waiting time before fractionation and biobank storage time.

**Figure S4.** Odds ratios versus *P*-values (“smile plots”) for metabolite univariate risk models by menopausal and fasting status.


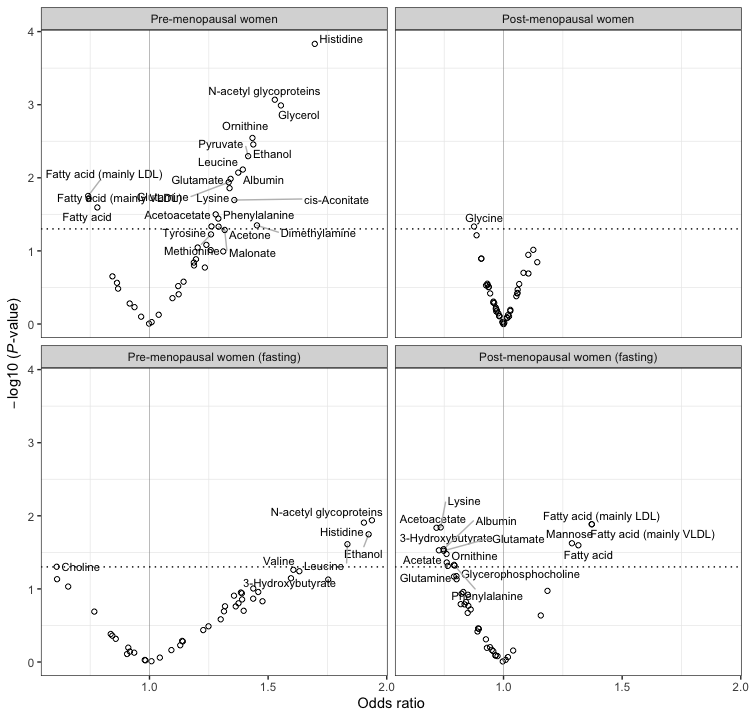


Dotted lines represent the raw *P*-value threshold of 0.05. No metabolites met the false discovery rate threshold in this analysis.
